# Genetic Dissection Uncovers Genome-Wide Marker-Trait Associations for Plant Growth, Yield, and Yield-Related Traits Under Varying Nitrogen Levels in Nested Synthetic Wheat Introgression Libraries

**DOI:** 10.3389/fpls.2021.738710

**Published:** 2021-10-04

**Authors:** Nitika Sandhu, Amandeep Kaur, Mehak Sethi, Satinder Kaur, Varinderpal-Singh, Achla Sharma, Alison R. Bentley, Tina Barsby, Parveen Chhuneja

**Affiliations:** ^1^School of Agricultural Biotechnology, Punjab Agricultural University, Ludhiana, India; ^2^Department of Soil Science, Punjab Agricultural University, Ludhiana, India; ^3^Department of Plant Breeding and Genetics, Punjab Agricultural University, Ludhiana, India; ^4^Genetics and Plant Breeding, National Institute of Agricultural Botany, Cambridge, United Kingdom; ^5^International Wheat and Maize Improvement Center (CIMMYT), Texcoco, Mexico

**Keywords:** GWAS, MTAs, Nitrogen, SNP markers, Wheat, Yield, synthetic wheat introgression lines

## Abstract

Nitrogen is one of the most important macronutrients for crop growth and metabolism. To identify marker-trait associations for complex nitrogen use efficiency (NUE)-related agronomic traits, field experiments were conducted on nested synthetic wheat introgression libraries at three nitrogen input levels across two seasons. The introgression libraries were genotyped using the 35K Axiom^®^ Wheat Breeder's Array and genetic diversity and population structure were examined. Significant phenotypic variation was observed across genotypes, treatments, and their interactions across seasons for all the 22 traits measured. Significant positive correlations were observed among grain yield and yield-attributing traits and root traits. Across seasons, a total of 233 marker-trait associations (MTAs) associated with fifteen traits of interest at different levels of nitrogen (N0, N60, and N120) were detected using 9,474 genome-wide single nucleotide polymorphism (SNP) markers. Of these, 45 MTAs for 10 traits in the N0 treatment, 100 MTAs for 11 traits in the N60 treatment, and 88 MTAs for 11 traits in the N120 treatment were detected. We identified putative candidate genes underlying the significant MTAs which were associated directly or indirectly with various biological processes, cellular component organization, and molecular functions involving improved plant growth and grain yield. In addition, the top 10 lines based on N response and grain yield across seasons and treatments were identified. The identification and introgression of superior alleles/donors improving the NUE while maintaining grain yield may open new avenues in designing next generation nitrogen-efficient high-yielding wheat varieties.

## Introduction

The global demand for nitrogen currently stands at about 117 million metric tons with a projected annual increase of approximately 1.5% expected in the near future (FAO, 2019). Farmers generally apply high doses of nitrogenous fertilizers to ensure good yields. The high input of commercially available fertilizers has led to the degradation of air, soil, and water quality (Hickman et al., 2014; Russo et al., 2017). In addition, when the supply of nitrogen (N) is in excess of crop N demand, it increases the susceptibility of plants to various diseases and insect pests (Reddy, 2017). Therefore, it is necessary to optimize and improve the nitrogen use efficiency (NUE) of cereal crops to maximize yield in addition to minimizing the negative impact of increase in N use on the environments and natural resources. Identification of marker-trait associations (MTAs) can be used to make effective targeted introgressions and is one possible genetic method to address the challenge of developing N-efficient wheat varieties with stable yield under N-limited environments.

Wheat varieties that maintain yield under moderate or intense N deficiency can adapt to low input systems. To breed such varieties, genetic variation for adaptation traits to N deficiency is required. To date, limited quantitative trait loci (QTL) for both yield and its response to N deficiency in wheat under field conditions have been documented. Detection of genotypes and underlying QTLs for maintaining yields at low N levels are of value in wheat breeding programs designed to increase N-deficiency tolerance. Some QTLs influencing N uptake have been genetically mapped in wheat under different doses of fertilizer application using biparental populations (An et al., 2006; Laperche et al., 2008; Xu et al., 2014; Mahjourimajd et al., 2016; Deng et al., 2017). A number of genetic loci for agronomic traits related to N use and grain yield have also been mapped to the chromosomal regions containing the *GS2* gene in wheat and rice (Prasad et al., 1999; Yamaya et al., 2002; Obara et al., 2004; Habash et al., 2007; Laperche et al., 2008; Fontaine et al., 2009). This suggests the role of the genomic region surrounding *GS2* (Pritchard and Wen, 2004) is favorable in breeding wheat and rice varieties with improved agronomic performance and nitrogen use efficiency (NUE). Other genetic regions associated with N uptake have also been detected in rice (Wissuwa et al., 1998; Ming et al., 2000), wheat (Su et al., 2006, 2009); maize (Zhu et al., 2005), common bean (Liao et al., 2004; Yan et al., 2004), and soybean (Li et al., 2005; Liang et al., 2010). The *NRT2.1, NRT2.2*, and *NAR2.1* genes have been reported to be the important contributors to the high-affinity transport system in *Arabidopsis* roots (Orsel et al., 2006). Sixteen genes were identified in wheat homologous to characterize the low-affinity nitrate transporter *NPF* genes in *Arabidopsis*, suggesting a complex wheat *NPF* gene family (Buchner and Hawkesford, 2014). The regulation of wheat *NPF* genes by plant N-status indicated the involvement of these transporters in the substrate transport in relation to N-metabolism.

The phenotypic traits reported to be associated with NUE in cereal crops so far include root number, length, density, and branching (Morita et al., 1988; Yang et al., 2012; Steffens and Rasmussen, 2016), dense and erect panicle (Sun et al., 2014), plant height (Gaju et al., 2011), and leaf width (Zhu et al., 2020). The collocation of QTLs for N-uptake and root architecture traits have suggested that breeding for better and efficient root systems is a way to improve NUE (Coque et al., 2008; Sandhu et al., 2015).

Diverse accessions, landraces, breeding populations, and next-generation mapping populations, including nested-association mapping (NAM) and multi-parent advanced generation inter-cross (MAGIC) populations have shown a potential for mining novel genetic variation in rice (Zhao et al., 2011; Sandhu et al., 2019; Subedi et al., 2019), wheat (Mackay et al., 2014), maize (Yu et al., 2008), and soybean (Xavier et al., 2015). The NAM and MAGIC populations have proven advantageous over biparental populations as they capture additional recombination breakpoints, thus increasing the allelic diversity and improving the power of QTL detection (Yu et al., 2008; Scott et al., 2020). Further, the availability of high throughput genotyping platforms to generate uniformly distributed genome-wide molecular markers is critical for the high-resolution genetic dissection of polygenic traits, and the tracking of favorable alleles in breeding populations (Pandey et al., 2012, 2016; Varshney et al., 2013). To date, a series of high-density wheat single nucleotide polymorphism (SNP) arrays, such as the Illumina 9K iSelect SNP array (Cavanagh et al., 2013), Illumina 90K iSelect SNP genotyping array (Wang et al., 2014a), 15K SNP array (Boeven et al., 2016), Axiom® 660K SNP array, 55K SNP array, Axiom® HD 820K genotyping array (Winfield et al., 2016), 35K Axiom array (Allen et al., 2017), and 50K Triticum TraitBreed array (Rasheed and Xia, 2019) have been developed and their utility has been demonstrated across a range of applications.

In the present study, we developed nested synthetic wheat introgression libraries capturing novel genetic variation. The libraries were genotyped using a high-density SNP array and phenotypically assessed for root traits and agronomic performance under three N input conditions in the field. Genome-wide association mapping was used to identify MTAs for the root and agronomic traits, and lines carrying favorable genetic combinations were also identified for use in future breeding for improved N use.

## Materials and Methods

### Plant Material

**Figure 1:**
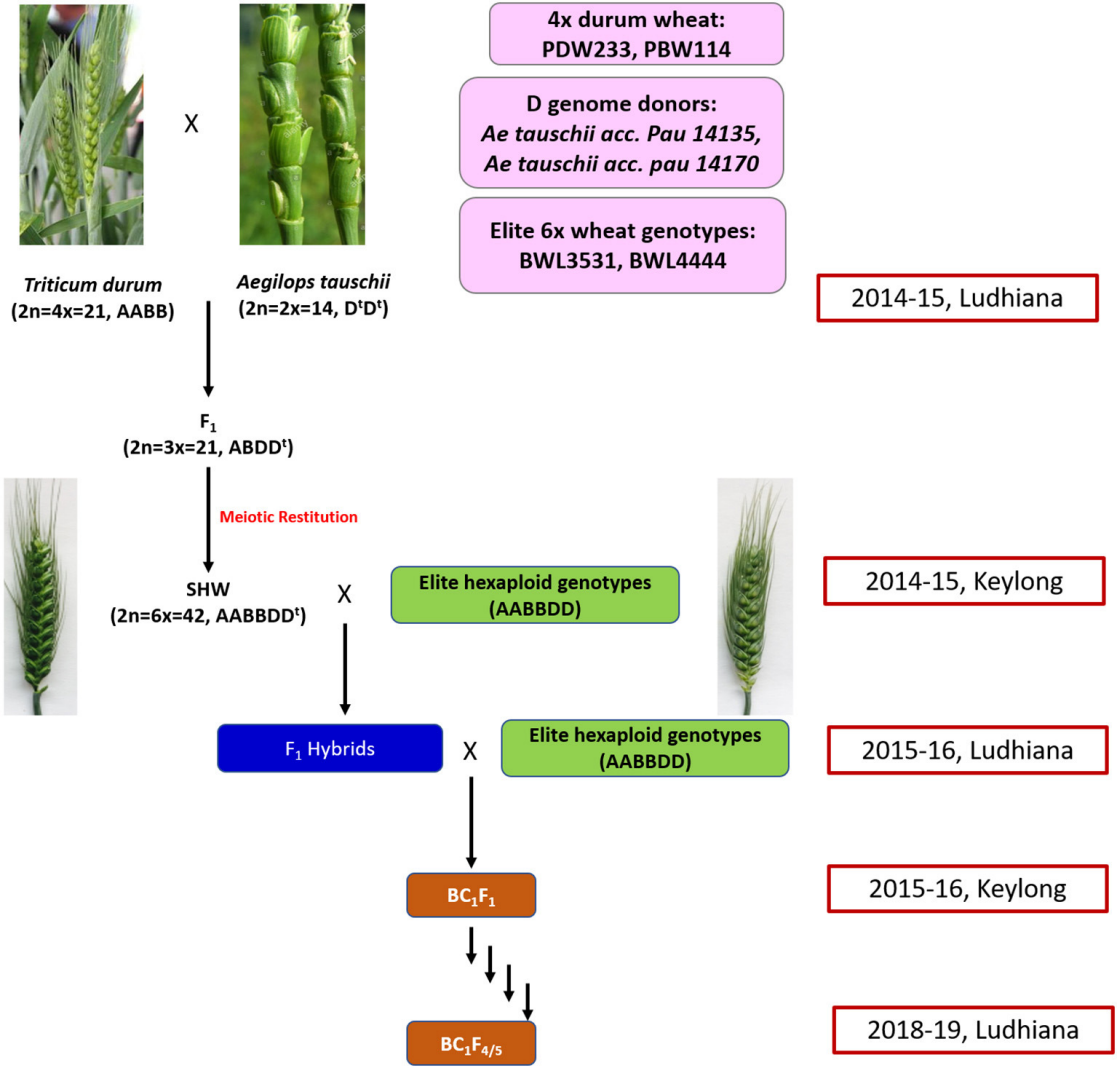
Schematic representation of the breeding strategy that is used to develop the nested synthetic wheat introgression libraries.

A total of 31 cultivated and 12 synthetic wheats were evaluated at 6 nitrogen (N) levels (N0, N40, N80, N120, N160, and N200) in 3 replications in 2015 and 2016 during the rabi seasons at Punjab Agricultural University, Ludhiana, India. The synthetic wheats, PDW233/*Ae. tauschii* acc. pau 14135 and PBW114/*Ae. tauschii* acc. pau 14170 produced high-grain yields as well as high agronomic efficiency at low fertilizer N doses (unpublished data). Nested introgression line libraries developed from the synthetic wheats, PDW233/*Ae. tauschii* acc. pau 14135 and PBW114/*Ae. tauschii* acc. pau 14170 (N-SHW) constituting a set of 352 lines were used in the present study. The N-SHW library was made up of subsets from four populations (Pop1: 75 lines from PDW233/*Ae. tauschii* acc. pau 14135 amphiploid//2^*^BWL4444; Pop2: 106 lines from PDW233/*Ae. tauschii* acc. pau 14135 amphiploid//2^*^BWL3531; Pop3: 88 lines from PBW114/*Ae. tauschii* acc. pau 14170 amphiploid//2^*^BWL4444; Pop4: 83 lines from PBW114/*Ae. tauschii* acc. pau 14170 amphiploid//2^*^BWL3531 along with the two common parents (BWL3531, BWL4444) and other unique parents (PDW233, PBW114, *Ae. tauschii* acc. pau 14135 amphiploid, and *Ae. tauschii* acc. pau 14170 amphiploid). The breeding scheme that was used to develop the N-SHW introgression library is summarized in Figure 1.

#### Agronomic Practices and Management of Experiments

The N-SHW library, six parents, and two synthetic hexaploid wheats were assessed under field conditions at the experimental farms of School of Agricultural Biotechnology, PAU Ludhiana (30 54' N latitude, 75 48' E longitude, and 247 m above sea level) over 2 years in 3N levels (6-year x N combinations). The soil analysis of experimental plots showed soil pH: 7.3; EC: 0.157 ds/m; organic content: 0.29%; N: 99 Kg ha^−1^, P: 69 Kg ha^−1^; K: 116 Kg ha^−1^, Zn: 4.77 parts per million (ppm), Cu: 1.73 ppm, and Fe: 7.30 ppm. The N-SHW libraries were evaluated in *rabi* seasons of 2018 and 2019. Details of the number of lines tested and experimental design is provided in Table 1. The breeding material was sown on 21^st^ of November and 18^th^ of November in 2018 and 2019, respectively. In both the years, the experiments were conducted at three N levels [i.e. zero N (0 Kg ha^−1^), half N (60 Kg ha^−1^), and full N (recommended, 120 Kg ha^−1^),] referred to as N0, N1, and N2, respectively. The recommended dose of phosphorus, potassium, and manganese was applied at the time of sowing. Half of N was applied at the time of sowing while the other half was applied in two equal splits, the first at crown root initiation stage and the remaining at the maximum tillering stage in both the N1 and N2 experiments. N0 was treated as a control. Recommended fungicides and insecticides were applied to control stripe rusts, brown rusts, and aphids at jointing, booting, and 10 days after anthesis to prevent diseases and pests. Weeds were controlled manually.

**Table 1:** Details on experimental material evaluated for nitrogen use efficiency related traits and design used across two crop seasons (2018–2019 and 2019–2020).

**Pop**	**Pedigree**	**Total no lines**	**Design**
Pop1	PDW233-*Ae. tauschii* acc. pau14135 amphiploid//BWL4444	75	Augmented design in 2018–2019 season; Split plot design considering nitrogen level main plot, breeding lines as subplots, 2 replications in 2019–2020 season, 2 rows plot (1.5 m long with 20 cm row to row spacing) in both 2018–2019 and 2019–2020 seasons.
Pop2	PDW233-*Ae. tauschii* acc. pau 14135 amphiploid//BWL3531	106	Augmented design in 2018–2019 season; Split plot design considering nitrogen level main plot, breeding lines as subplots, 2 replications in 2019–2020 season, 2 rows plot (1.5 m long with 20 cm row to row spacing) in both 2018–2019 and 2019–2020 seasons.
Pop3	PBW114-*Ae. tauschii* acc. pau 14170 amphiploid//BWL4444	88	Split plot design considering nitrogen level main plot, breeding lines as subplots, 2 replications, 1.5 m x 2 rows plot in both 2018–2019 and 2019–2020 seasons.
Pop4	PBW114-*Ae. tauschii* acc. pau 14170 amphiploid//BWL3531	83	Split plot design considering nitrogen level main plot, breeding lines as subplots, 2 replications, 1.5 m x 2 rows plot in both 2018–2019 and 2019–2020 seasons.

#### Characterization of Phenotypic Traits

Under field conditions, a total of 22 traits were assessed in all experiments across both seasons except the maximum root length and root angle which were measured in 2018 only. The details of the nitrogen use efficiency (NUE)-related traits, root and plant morphological traits, grain yield, and yield attributing traits are presented in Supplementary Figure 1. Destructive sampling of six plants per plot was done at 60 days after sowing (DAS) to evaluate early root and shoot traits (Supplementary Figure 2). Shoots were separated from the roots, and the fresh root weight (FRW; g) and fresh shoot weight (FSW; g) were measured. The root and shoot samples were dried at 70°C in an oven until constant dry root weight (DRW; g) and dry shoot weight (DSW; g) were observed, while the roots were cleaned thoroughly and stored in 70% alcohol at 4°C for root trait evaluation. The maximum root length (MRL) and root angle (RA) were measured using ImageJ software. total root length (TRL), total root surface area (RSA), total root diameter (RD), total root volume (RV), number of forks (NF), and number of tips (Ntips) were recorded using WinRhizo STD4800 (Supplementary Figure 2). The roots were then dried at 70°C in the oven until constant DRW was observed. The data on N-uptake related traits were recorded using a chlorophyll meter, Soil Plant Analysis Development Meter (SPAD502) and leaf color chart (LCC). The LCC provides a decision-support system to the farmers for sustaining the high yields with optimum N dose in the field crops. It measures the leaf color variations of 6 SPAD units comprising 3, 3.5, 4.0, 4.5, 5.0, and 6.0 and provides N recommendation in the field crops. Flag leaf length (FLL) and flag leaf width (FLW) were recorded using a centimeter scale. Days to flowering (DTF) rate of about 50% was recorded when 50% of the plants in a plot exerted their panicles. Spikelets per spike (SPS) were counted manually from five random plants. The number of productive tillers (NPT) was counted manually in 0.5 m row length and shoot biomass (SB) at harvesting was measured from 0.5 m row length. The plant height (PHT) in cm was measured as the mean height of five random plants for each entry measured from the base of the plant to the tip of the panicle at the maturity stage. The plants were harvested at physiological maturity or when 80–85% of the panicles turned to golden yellow and the panicles at the base were already at the hard dough stage; harvested grains were threshed, dried, and weighed to determine the grain yield (GY). The shoot biomass (SB) was recorded at harvesting.

#### Phenotypic Data Analysis

Analysis of variance, and experiment-wise mean for each season was calculated using a mixed model analysis in PBTools V 1.4.0. for augmented design and in STAR Version: 2.0.1 for the split plot design. In the split plot design, the N levels were considered as the main plot and the breeding lines as subplot. Fisher's *t*-test was used to determine the significant difference among the breeding lines, treatments, and to estimate the interactions. The correlation analysis among traits was performed in R. v.1.1.423.

To evaluate the phenotypic stability and GY adaptability of the breeding lines across seasons and treatments, the genotype and genotype × environment (GGE) biplot analysis was performed, considering the effects of genotype (G) and genotype by environment (GE) as random. The best linear unbiased prediction (BLUP) values of the G and GE effects were calculated. The multiplicative model in PB tool version 1.3 (http://bbi.irri.org/) was used to explain the relationship between G and the seasons.

### Genotypic Data

High-density genotyping was performed using the 35K Axiom^®^ Wheat Breeder's Array (Affymetrix UK Ltd., United Kingdom). The quality pre-processing of 35,143 markers obtained from the 35K chip was done using PLINK software (Purcell et al., 2007). A total of 9,474 single nucleotide polymorphisms (SNPs) with minor allele frequency (MAF) of >5%, maximum heterozygote proportion of 0.1 and missing rates <0.1 were used to estimate the genetic relationships and for the mapping of MTAs for different traits associated with plant growth, yield, and yield-related traits. Principal component analysis (PCA) was carried out to detect and correct for population structure.

#### Population Structure and Association Analysis

The model-based STRUCTURE V. 2.3.4 software was used to test *K* values from 1 to 10, with a burn-in period of 10,000 and 1,000,000 Markov chain Monte Carlo (MCMC) reps after burn-in in order to assess the population structure in the 352 breeding lines using a total of 9,474 SNPs. The consistency and accuracy of the results were validated across 10 runs for each *K*. The *K* value with maximum likelihood over the 10 runs was used to estimate the most appropriate number of clusters (Pritchard and Wen, 2004). The population structure was determined by plotting the proposed number of subpopulations against the delta *k* (Earl, 2012). The PCA was performed in Genome Association and Prediction Integrated Tool (R/GAPIT) and added iteratively to the fixed model, ranging from PC1 to PC10.

Significant marker-trait associations were identified using a compressed mixed linear model (CMLM)/P3D (population parameters previously defined) in GAPIT executed in R. Identity by state (IBS) values and a relatedness matrix were used to estimate the random effect and genetic similarity of the accessions, respectively. The statistical power of the association studies was further improved by considering the population structure (*Q* value) and kinship matrix (*K*) estimated from the genotyping data. The Bonferroni correction method was used to correct for false positives in the analysis, using the stringent *p*-value benchmark. The Bonferroni multiple test correction was performed (0.05/9474; significance level of 5%/total number of markers used in analysis) and the calculated threshold value was found to be 5.28 × 10^−6^. The allelic effect of all the significant markers associated with the measured traits was determined by comparing the mean phenotypic values and the significant allelic variants for the trait/s using a Kruskal–Wallis test in R.

#### Candidate Gene Analysis and Functional Annotation of Putative Candidate Genes

Single nucleotide polymorphisms that exhibited a false discovery rate (FDR) corrected *p* < 0.05 for a particular trait of interest were evaluated as markers for the potential putative candidate genes. A window of 1 Mb adjacent to each significant SNP was examined for candidate genes and annotations were identified through the Ensembplants database (http://plants.ensembl.org/index.html).

The functional annotation and gene ontology (GO) of the identified putative candidate genes were performed using OMIX box software. Blast (*E* ≤ 1 0^−5^) was performed using the CloudBlast tool against *Triticum* (nr_subset) [monocots_*triticum*, taxa:4564] and NCBI non-redundant database (http://www.ncbi.nlm.nih.gov), followed by the InterPro using CloudIPS, followed by GO mapping, and annotation configuration. The GO terms were then used to generate the semantic similarity-based scatterplots/interactive graphs/tag clouds by using REVIGO (http://revigo.irb.hr/).

### Defining N-Irresponsive and N-Responsive Lines

The genotypes that showed more or equal/stable GY with the minimal application of nitrogen fertilizer when compared to the recommended or standard N fertilizer application were considered as the N irresponsive (NIR) genotypes or the top grain yielders across seasons and treatments. On the other way around, the genotypes that were low yielding or not able to maintain the GY with the minimal application of N fertilizer when compared to the recommended or standard N fertilizer application, were considered as the N responsive (NR) genotypes or the poor grain yielders across seasons and treatments.

## Results

### Significant Phenotypic Trait Variation and Correlations Detected Across Nitrogen Treatments

The 352 N-SHW lines, six parents, and two synthetic hexaploid wheat donors were screened for 22 traits in six growing conditions (2 years x 3 nitrogen levels). The ANOVA revealed significant genetic variation for the root, plant morphological, and agronomic traits among genotypes, treatments, seasons, and their interactions (genotype x treatment, genotype x season, treatment x season and genotype x treatment x season) (Table 2). The detailed information on trial means, least significance difference (LSD), and heritability for all the traits measured are presented in Supplementary Table 1. The results revealed significant genetic variations across genotypes, treatments, and their interactions in 2018–2019 and 2019–2020 seasons for all the traits measured (Supplementary Table 2). The phenotypic data of the traits measured in the present study were averaged across two seasons and are presented as mean values in Supplementary Table 3.

**Table 2:** Analysis of variance (ANOVA) for the NUE-related, root, plant morphological, yield, and yield-related traits among genotype (G), treatments (T), seasons (S), and their interactions (G x T, genotype x treatment; G x S, genotype x season; T x S, treatment x season; and G x T x S, genotype x treatment x season).

**Population**	**Source of variation**	**DF**	**LCC**	**SPAD**	**FRW**	**DRW**	**FSW**	**DSW**	**DTF**	**NPT**	**PHT**	**SPS**	**SB**	**FLL**	**FLW**	**GY**	**TRL**	**RSA**	**RD**	**RV**	**Tips**	**Forks**
			***F*** **-value**																			
PDW233/*Ae. tauschii* 14135 amphiploid//BWL4444	G	74	7.45^***^	4.47^***^	2.44^***^	1.89^***^	5.84^***^	7.2^***^	58.79^***^	3.22^***^	4.9^**^	4.92^*^	2.27^***^	35.01^***^	88.48^***^	5.14^***^	1.56^**^	3.90^*^	0.872	3.81^*^	8.84^***^	3.13^***^
	T	2	244.8^***^	286.5^***^	27.12^***^	56.14^***^	88.41^***^	55.36^***^	141.44^***^	254.3^***^	4.26^*^	5.29^**^	56.37^***^	120.87^***^	261.42^***^	14.18^***^	73.78^***^	27.41^***^	48.15^***^	6.5^**^	125.1^*^	86.92^***^
	S	1	612.8^***^	637.2^***^	117.9^***^	18.49^***^	447.45^***^	394.06^***^	605.42^***^	30.88^***^	4.04^*^	2.08^*^	7.22^**^	241.96^***^	441.38^***^	109.4^***^	124.8^***^	4.93^*^	90.05^***^	1.269^*^	1,062^***^	277.8^***^
	G x T	148	4.60^***^	2.19^***^	1.78^***^	1.62^***^	2.07^***^	2.76^***^	2.07^***^	3.49^***^	5.81^**^	3.88^*^	2.04^***^	1.65^***^	34.33^***^	1.49^**^	1.31^*^	2.99^*^	0.672	1.87^*^	3.70^***^	1.71^***^
	G x S	74	7.38^***^	3.2^***^	3.61^***^	1.8^***^	3.28^***^	3.79^***^	10.34^***^	1.84^***^	5.04^*^	3.07^*^	1.74^**^	1.84^***^	53.87^***^	1.798^***^	1.32^*^	2.72^*^	0.205	1.41^*^	9.90^***^	3.37^***^
	T x S	2	3.24^*^	10.53^***^	8.45^***^	8.22^***^	130.87^***^	56.21^***^	68.42^***^	44.65^***^	6.07^**^	2.81^*^	116.6^***^	42.47^***^	572.6^***^	20.84^***^	3.69^*^	4.45^**^	21.75^***^	3.212^*^	22.46^***^	11.50^***^
	G x T x S	148	6.62^***^	2.06^***^	1.855^***^	1.12	2.78^***^	3.21^***^	2.98^***^	1.94^***^	6.45^**^	4.44^**^	1.88^***^	5.92^*^	67.56^***^	1.53^**^	1.19^*^	2.64^*^	0.397	1.47^*^	5.95^***^	1.87^***^
PDW233/*Ae. tauschii* 14135 amphiploid//BWL3531	G	105	6.83^*^	4.8^*^	1.45^**^	4.58^***^	2.45^***^	2.87^***^	60.11^***^	2.76^***^	3.65^***^	7.15^***^	2.20^***^	15.97^***^	106.4^***^	4.93^***^	3.73^***^	1.38^*^	4.15^***^	2.76^*^	5.02^***^	3.94^***^
	T	2	1.899^*^	3.5^*^	2.94^**^	5.10^**^	53.34^***^	41.31^***^	188.7^***^	401^***^	135.5^***^	3.75^**^	74.41^***^	83.24^***^	420.3^***^	62.89^***^	44.36^***^	9.05^***^	65.06^***^	2.13^*^	82.64^***^	53.1^***^
	S	1	5.14^*^	5.5^*^	25.45^***^	81.57^***^	420.24^***^	1089.9^***^	14,660^***^	1.76^*^	139.8^***^	26.57^***^	3.12^**^	13,821^***^	89,346^***^	62.44^***^	1,809^***^	334^***^	242.5^***^	2.03^*^	2,244^***^	1,871^***^
	G x T	210	4.82^*^	4.8^*^	1.52^***^	4.98^*^	1.26^*^	1.49^***^	2.33^***^	2.35^***^	2.31^***^	3.26^*^	1.63^***^	1.27^*^	2.96^***^	1.71^***^	1.41^*^	2.99^*^	3.54^*^	2.82^*^	2.05^***^	1.40^**^
	G x S	105	4.36^*^	3.3^*^	1.71^***^	3.73^*^	1.37^*^	1.29^*^	9.23^***^	1.91^***^	2.73^***^	3.11^*^	2.93^**^	1.34^*^	5.07^***^	1.84^***^	1.62^***^	2.61^*^	2.42^*^	3.31^*^	2.86^***^	2.04^***^
	T x S	2	3.06^*^	3.1^*^	17.76^***^	9.11^***^	36.98^***^	9.09^***^	126.58^***^	97.34^***^	2.74^*^	3.19^*^	193.4^***^	34.09^***^	976.9^***^	39.24^***^	279.4^***^	85.6^***^	39.58^***^	3.57^*^	268.6^***^	247^***^
	G x T x S	210	4.41^*^	4.4^*^	1.43^**^	4.70^*^	1.50^***^	1.43^*^	2.69^***^	1.25^*^	2.02^***^	2.99^*^	1.39^**^	1.68^*^	2.86^***^	1.79^***^	1.37^**^	3.62^*^	3.27^*^	2.41^*^	3.27^***^	1.93^***^
PBW114/*Ae. tauschii* 14170 amphiploid//BWL3531	G	87	2.24^***^	2.3^***^	1.94^***^	3.15^*^	1.42^*^	1.63^**^	3.40^***^	1.60^**^	3.22^***^	1.45^**^	1.54^**^	1.65^***^	1.49^***^	1.81^***^	2.01^***^	1.31^*^	11.06^***^	2.78^*^	2.15^*^	1.66^***^
	T	2	259.26^***^	297.6^***^	51.44^***^	14.33^***^	9.25^***^	4.96^*^	58.11^***^	267.6^***^	231.5^***^	19.11^***^	224.3^***^	398.9^***^	220.0^***^	61.77^***^	17.10^***^	30.7^***^	120.7^***^	33.84^***^	13.21^***^	14.9^***^
	S	1	42.69^***^	2.94^*^	565.7^***^	2.69^*^	11.69^***^	248.86^***^	8439.4^***^	1.24^*^	98.01^***^	893^***^	59.26^***^	1,026^***^	1,364^***^	2.10^*^	1,122^***^	516^***^	653.7^***^	4.69^*^	449^**^	682^***^
	G x T	174	2.65^***^	2.58^***^	2.22^***^	1.35^**^	1.43^**^	1.47^**^	3.78^***^	1.67^***^	2.80^***^	1.75^*^	1.34^*^	1.26^*^	1.68^***^	1.74^***^	2.46^***^	1.37^**^	2.94^*^	2.94^*^	1.42^**^	1.79^***^
	G x S	87	2.55^***^	3.01^***^	2.0^***^	1.50^**^	1.78^*^	1.43^*^	2.70^***^	2.03^***^	3.99^***^	1.74^*^	1.54^*^	1.79^***^	2.04^***^	1.49^*^	1.80^***^	0.97	2.94^*^	3.67^*^	1.99^*^	1.58^**^
	T x S	2	90.59^***^	95.61^***^	51.14^***^	13.09^***^	78.71^***^	18.81^***^	13.57^***^	163.8^***^	21.45^***^	10.34^***^	69.76^***^	21.72^***^	584^***^	17.82^***^	43.22^***^	4.05^*^	120.5^***^	25.83^***^	31.73^***^	20.1^***^
	G x T x S	174	2.10^***^	2.14^***^	1.89^***^	1.25^*^	1.53^***^	1.80^*^	3.75^***^	1.58^***^	3.32^***^	1.26^*^	1.26^*^	1.41^**^	1.65^***^	1.71^***^	2.43^***^	1.36^**^	2.94^*^	2.90^*^	1.46^**^	1.79^***^
PBW114/*Ae. tauschii* 14170 amphiploid//BWL4444	G	82	2.05^*^	3.0^**^	1.54^*^	2.99^*^	2.13^*^	1.94^*^	1.47^**^	1.29^*^	1.99^*^	1.32^*^	1.68^***^	1.54^*^	1.64^***^	1.87^*^	1.48^**^	1.98^*^	1.15	1.99^*^	1.54^**^	1.56^**^
	T	2	2.81^*^	2.96^*^	2.1^*^	1.85^*^	1.62^*^	2.84^**^	2.11^*^	1.61^*^	1.81^*^	1.28^*^	1.82^*^	2.08^*^	1.21^*^	1.34^*^	1.26^*^	2.21^*^	0.21	2.35^*^	1.99^*^	1.63^*^
	S	1	1.96^*^	2.68^*^	2.07^*^	1.85^*^	11.68^***^	3.67^**^	1.87^***^	2.93^*^	2.29^*^	1.30^*^	1.28^*^	2.01^***^	2.07^***^	2.87^*^	2.04^*^	1.87^*^	0.38	1.44^*^	2.23^**^	1.63^*^
	G x T	164	2.03^*^	2.33^*^	1.99^*^	2.28^*^	2.15^*^	1.89^*^	1.55^***^	1.45^***^	1.98^***^	1.49^***^	1.66^***^	1.83^***^	1.81^***^	3.95^*^	1.22^*^	1.98^*^	1.08	1.09^*^	1.27^*^	1.23^*^
	G x S	82	206^*^	3.07^*^	1.98^*^	2.67^**^	3.03^**^	3.72^**^	2.45^*^	1.21^*^	1.33^*^	2.83^*^	2.81^**^	2.33^*^	1.29^*^	1.99^*^	2.97^*^	2.87^*^	0.87	2.83^**^	1.64^*^	1.84^*^
	T x S	2	1.50^*^	3.46^**^	3.68^*^	1.64^*^	1.72^*^	1.61^*^	1.27^*^	2.36^*^	3.2^*^	2.02^*^	1.44^*^	2.03^*^	2.05^*^	2.82^*^	2.99^*^	1.63^*^	1.33	1.55^*^	1.57^*^	1.99^*^
	G x T x S	164	3.01^*^	2.98^*^	2.78^*^	2.94^**^	2.97^*^	1.74^*^	2.46^*^	2.77^*^	2.65^*^	2.79^*^	1.80^*^	2.25^*^	2.27^*^	2.91^*^	1.73^*^	2.62^*^	0.62	1.65^*^	1.76^*^	1.75^*^

* *significant at < 0.05 level*,

** *significant at < 0.01 level*,

*** *significant at < 0.001 level*.

Grain yield increased with applied N level. In the N0 treatment, the average GY of the tested breeding lines across seasons was 2,022 kg ha^−1^ and it ranged from 564 to 4,092 kg ha^−1^ (Supplementary Table S3). In the N60 treatment, the GY varied from 882 to 4,685 kg ha^−1^ with an average GY of 2,357 kg ha^−1^ and, while in N120 treatment, the GY varied from 1,332 to 4,270 with an average of 2,579 kg ha^−1^ (Supplementary Table 3). Across seasons, N in the limited conditions (N0) resulted in the 14% and 22% GY reduction compared to N60 and N120 treatments, respectively. The N application also significantly increased the shoot biomass (SB) by 8% in N60 and 52% in N120 treatment across seasons. The average NPT across experiments was higher in N120 (28) compared to N60 (24) and N0 (22) (Supplementary Table 3). Under the N0 treatment, the average value of the leaf color chart (LCC) varied from 3.3 to 4.8, from 3.5 to 5.1 in the N60 treatment, and from 4.1 to 5.3 in the N120 treatment (Supplementary Table 3). The response of lines in terms of average dry shoot weight (DSW) across seasons increased from 3.28 in N0 to 3.62 in N60 to 3.75 in N120 treatment (Supplementary Table 3). The minimum and maximum values of dry root weight (DRW) under N0 were 0.187 g and 2.425 g; 0.298 g and 2.001 g under N60, and 0.338 g and 2.333 g under N120, respectively (Supplementary Table 3). The average root diameter was the highest under N60 (0.610 g) compared to N0 (0.560 g) and N120 (0.409 g) (Supplementary Table 3). Across seasons, the average flowering was delayed by 2 days under the N0 treatment compared to the N60 and N120 treatments. Average plant height (PHT) was lower (92 cm) in N0 compared to N60 (95 cm) and N120 (99 cm).

**Figure 2:**
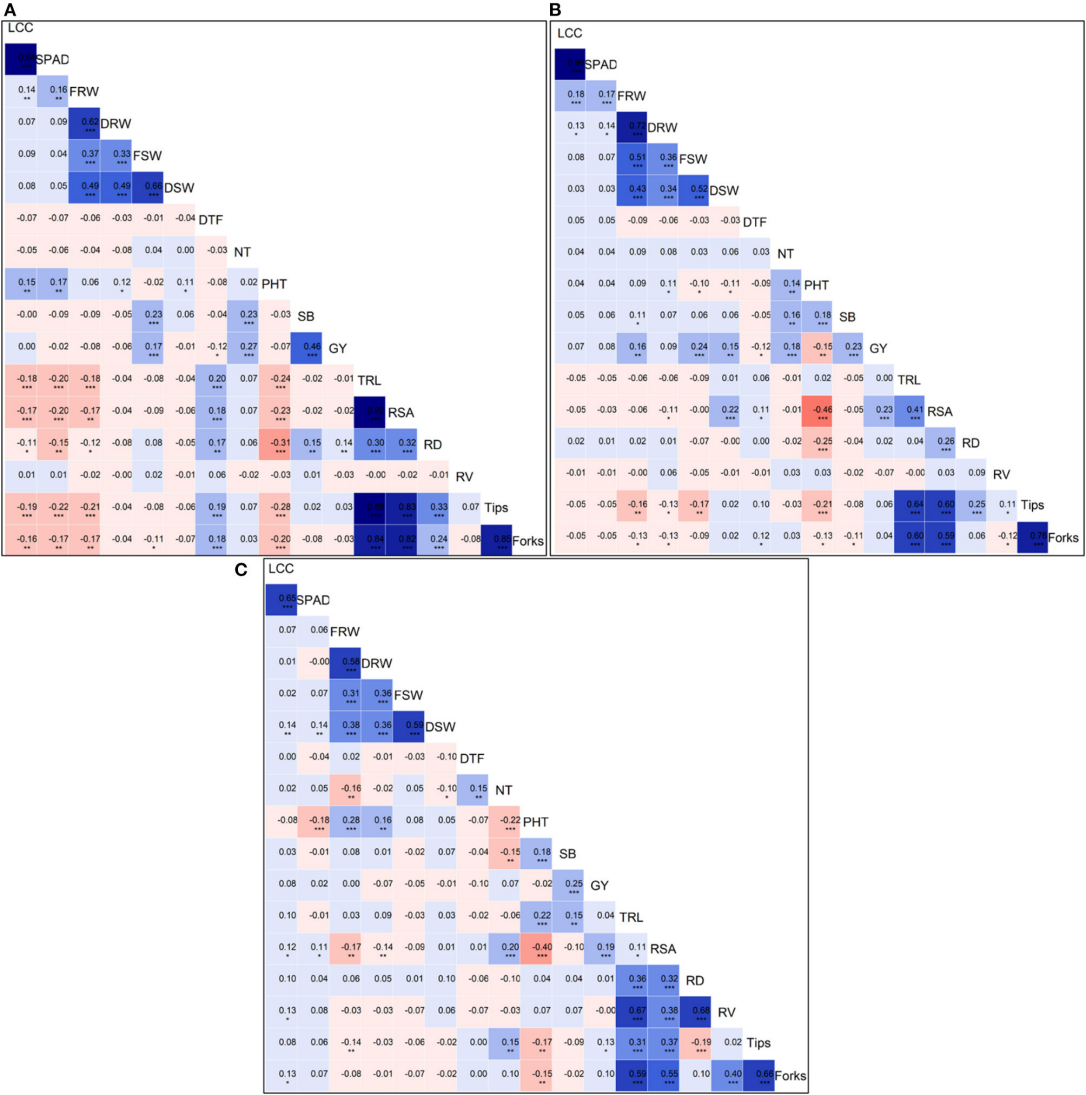
Plots of Pearson's *r-values* showing the correlation among traits measured **(A)** measured at N0, **(B)** N60, and **(C)** N120 levels. The blue color indicates positive correlation and red color indicates the negative correlation among different traits, the variation in color intensity represents the strength of the correlation among the traits. *Significance at <5% level, **significance at <1% level, ***significance at <0.1% level.

We calculated the Pearson's correlation coefficients between all the traits measured in N0 (Figure 2A), N60 (Figure 2B), and N120 (Figure 2C) treatments. The Pearson's correlation coefficients across all treatments considering pooled mean data for all the traits measured in the present study is presented in Supplementary Figure 3. The strongest and most significant positive correlation among grain yield and yield-attributing traits and root traits were observed in N60 treatment. The grain yield was significantly and positively correlated with shoot biomass (SB) (*r* = 0.23, *p* < 0.001), NPT (*r* = 0.18, p <0.01), fresh root weight (FRW) (*r* = 0.16, *p* < 0.01), fresh shoot weight (FSW) (*r* = 0.24, p <0.001), dry shoot weight (DSW) (*r* = 0.23, *p* < 0.001), and with root surface area (RSA) = (*r* = 0.23, *p* < 0.001). Across treatments, the GY showed a negative correlation with days to flowering (DTF), and shoot biomass (SB) showed a positive correlation with GY.

### Population Structure Analysis Detected Three Genetic Subpopulations

**Figure 3:**
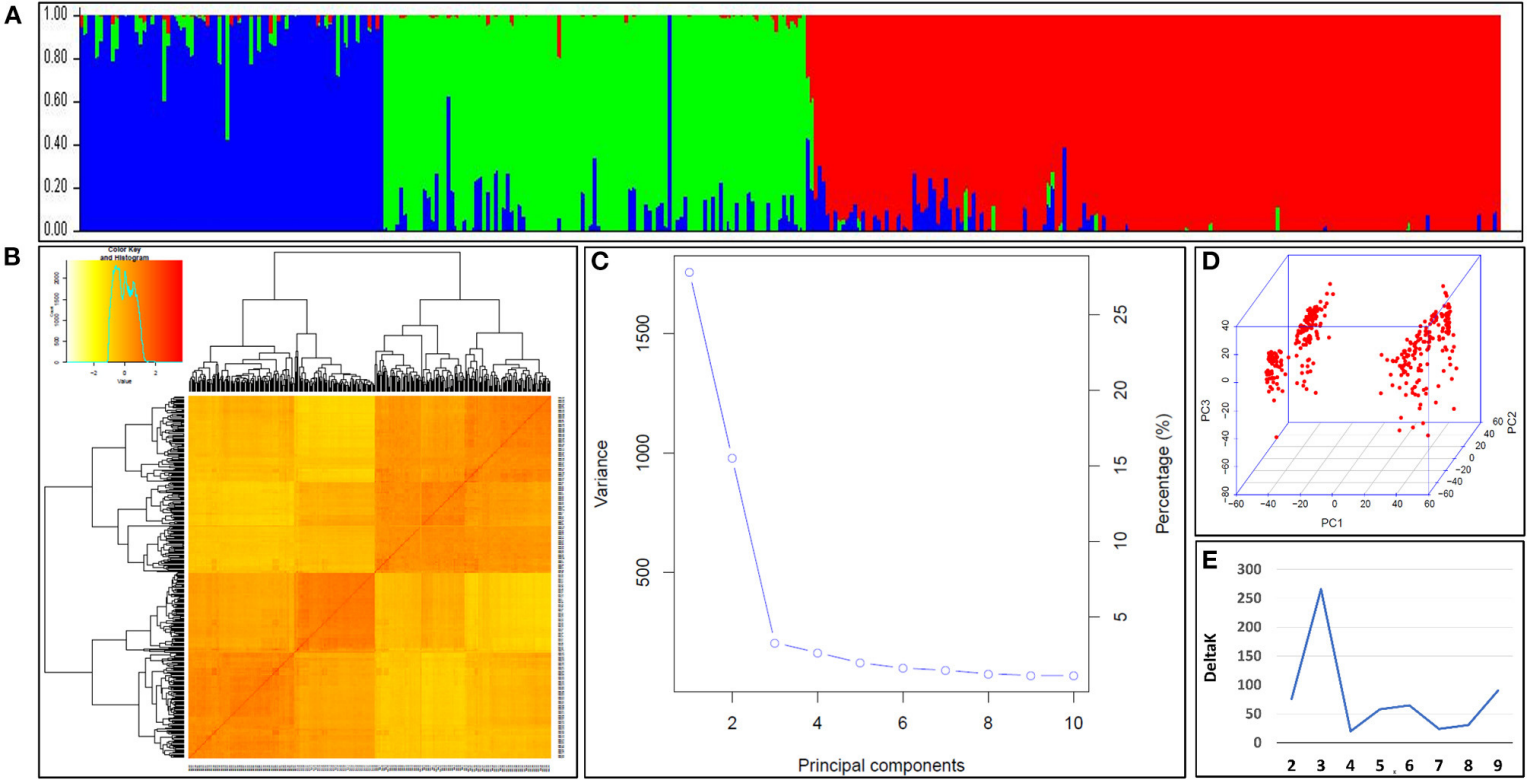
**(A)** Population structure within the nested synthetic wheat introgression libraries. The population structure plots with each vertical bar representing a breeding line colored according to the particular group to which the breeding line has been assigned. The breeding lines assigned to more than one of group represents the degree of their admixed set of the alleles **(B)** The Kinship matrix displayed as the heat map, where the red indicates the highest correlation between the pairs of breeding lines and yellow indicates the lowest correlation **(C)** The Scree plot indicating the most of the variabilities explained by the first three principal components (PCs) for association study **(D)** The three-dimensional view of the PCs explaining the genotypic variation among breeding lines constituting the introgression libraries. **(E)** The appropriate number of the subpopulations determined from the largest delta, *K* = 3.

The population structure of the N-SHW lines was assessed to understand the genetic structure of the 352 lines based on 9,474 single nucleotide polymorphisms (SNPs) distributed across all 21 wheat chromosomes. The most appropriate *K* explaining the population structure was *K* = 3 at minor allele frequency (MAF) ≥ 5% (Figure 3A). The kinship heatmap indicated a weak relatedness in the panel (Figure 3B). The first three principal components (PCs) were the most informative and gradually decreasing (Figures 3C,D) until the tenth PC. The kinship and PCs were considered during the genome-wide association study (GWAS) to correct for population structure. The appropriate number of subpopulations was determined from the largest *delta K* value of 3 (Figure 3E).

### Mapping Reveals Significant Marker-Trait Associations for All Traits

Genome-wide association study was performed exploiting the phenotypic variability in the 352 N-SHW lines using 9,474 SNPs from the 35K Axiom^®^ Wheat Breeder's Array. Using the -log(P) ≥0.001 at 5% significance level, a total of 233 marker-trait associations (MTAs) were detected across seasons associated with fifteen traits of interest at different N levels (N0, N60, and N120; Table 3). Of these, 45 MTAs were associated with 10 traits in the N0 treatment, 100 MTAs were associated with 11 traits in the N60 treatment, and 88 MTAs were associated with 11 traits in the N120 treatment (Table 3). Across seasons and N treatments, a total of 53 MTAs associated with more than one trait/treatment were detected (Table 3). In addition to these 53 MTAs, another 41 MTAs associated with a single trait only were detected across seasons (Supplementary Table 4). All MTAs detected in the present study either in one season, both seasons, each treatment, or across treatments are compiled in Supplementary Table 5. Mapping detected MTAs on all subgenomes (A: 42, B: 18, and D: 34) across seasons and treatments. The highest number of MTAs were detected on chromosome 6A (26) followed by 2D (25), 3B (12), 4A (9), 6D (8), 2A (6), and 2B (4) with 1 MTA on each of 1B, 4B, 5A, and 7D. Considering all assessed traits, significant MTAs were reported for fresh root weight (FRW) (2A, 2D), fresh shoot weight (FSW) (2A, 2B, 2D, 5A, 7A), dry root weight (DRW) (2A, 2B, 2D, 7A), dry shoot weight (DSW) (2A, 2B, 2D), flag leaf width (FLW) (4A, 4B), number of tillers (NT) (3A), spikelets per spike (SPS) (1B), days to flowering (DTF) (3A, 3B, 6A), shoot biomass (SB) (6A), total root length (TRL) (6D), root surface area (RSA) (6A, 6D), root volume (RV) (6D), tips (6A, 6D) and forks (6A, 6D).

**Table 3:** The significant marker-trait associations (MTAs) and putative candidate genes identified across different treatments for the NUE-related, root, plant morphological, yield, and yield-related traits in a genome wide association study (GWAS) conducted on nested synthetic wheat introgression libraries.

**SNP**	**Chr**	**Position (bp)**	**Trt/Trait**	***p-*value**	***R* ^**2**^**	**FDR**	**Gene stable ID**	**Gene end (bp)**	**Gene start (bp)**	**Description**	**Function**
AX-95136668	3A	690432670	N60, N120 (GY)	5.85E-07	0.202	0.001	TraesCS3A02G452300	690460596	690459736	Flowering-promoting factor 1-like protein 2	Regulates flowering (Kania et al., 1997) and gibberellin signaling pathway
AX-94415776	6A	28700804	N60, N120 (GY)	1.54E-06	0.2	0.001	ENSRNA050010223	28845847	28845775		
AX-94978974	6A	29876500	N60, N120 (GY)	1.20E-06	0.202	0.001	TraesCS6A02G056800	29879453	29877038	Putative disease resistance protein At3g14460	Defense response to fungus (Bianchet et al., 2019)
AX-94737868	6A	29876631	N60, N120 (GY)	6.07E-07	0.204	0.001					
AX-95210745	6A	29967076	N60, N120 (GY)	1.27E-06	0.202	0.001	TraesCS6A02G057000	29969466	29967087	Putative F-box protein At3g16210	Regulates gibberellin signaling (McGinnis, 2003); panicle and seed development in rice (Jain et al., 2007; Li et al., 2011)
AX-95631197	6A	30030973	N60, N120 (GY)	5.94E-07	0.206	0.001	TraesCS6A02G057100	30036467	30032329	F-box protein At5g03970-like	APO gene in rice improved grain yield per plant (Terao et al., 2010)
AX-95011132	6A	30031026	N60, N120 (GY)	7.51E-07	0.202	0.001					
AX-95255669	6A	30873212	N60, N120 (GY)	8.32E-07	0.202	0.001	TraesCS6A02G058500	30876811	30873143	L-type lectin-domain containing receptor kinase IX.1	Promotes cell death (Wang et al., 2015), resistance response to pathogens (Wang et al., 2014a)
AX-95219967	6A	31036496	N60, N120 (GY)	1.11E-06	0.201	0.001	TraesCS6A02G058700	31036808	31034388	LURP-one-like protein (DUF567)	Defense and resistance to *H. parasitica* mediated by the R-proteins RPP4 and RPP5 (Gallego-Giraldo et al., 2018)
AX-95070275	6A	31048271	N60, N120 (GY)	7.49E-07	0.204	0.001					
AX-94970334	6A	31474354	N60, N120 (GY)	1.30E-06	0.199	0.001					
AX-94894393	6A	34032797	N60, N120 (GY)	8.00E-07	0.201	0.001	TraesCS6A02G063700	34033068	34029127	F-box protein At5g03970-like	APO gene in rice improved grain yield per plant (Terao et al., 2010)
AX-95249202	6A	34285660	N60, N120 (GY)	8.46E-07	0.199	0.001	TraesCS6A02G064600	34285946	34283159	Predicted protein	
AX-94553503	6A	34982713	N60, N120 (GY)	8.83E-07	0.201	0.001	TraesCS6A02G065400	34983010	34979092	Serine/arginine-rich splicing factor RS41 isoform X2	Abiotic stress tolerance (Palusa et al., 2007)
AX-94696366	6A	35482343	N60, N120 (GY)	7.06E-07	0.201	0.001	TraesCS6A02G066200	35484242	35478280	Protein accumulation and replication of chloroplasts 6, chloroplastic	Chloroplast division (Zhang et al., 2015), regulation of mitochondrial DNA replication as well as gene transcription and translation (Tang et al., 2019)
AX-94386201	6A	35580470	N60, N120 (GY)	7.83E-07	0.2	0.001	TraesCS6A02G066800	35581719	35580136	Transcription termination factor MTERF4, chloroplastic-like	Chloroplast or mitochondria development (Quesada, 2016)
AX-94835065	6A	36106101	N60, N120 (GY)	1.46E-06	0.197	0.001	TraesCS6A02G067700	36110655	36105754	S-(+)-linalool synthase, chloroplastic-like	Monoterpene (C10) biosynthesis, resistance to the bacterial blight pathogen *Xanthomonas oryzae* pv. *Oryzae* (Taniguchi et al., 2014)
AX-94914391	6A	36429885	N60, N120 (GY)	1.17E-06	0.202	0.001	TraesCS6A02G068000	36433862	36429712	Putative HVA22-like protein g	Role in stress response (Brands and Ho, 2002), auxin transport to root tips (Janiak et al., 2016)
AX-94606161	6A	64319129	N60, N120 (GY)	1.37E-06	0.203	0.001	TraesCS6A02G097200	64320738	64317228	Phosphatidylinositol/phosphatidylcholine transfer protein SFH12-like	Transport of secretory proteins from the Golgi complex (Mo et al., 2007)
AX-95072891	6A	690432617	N60, N120 (GY)	1.47E-06	0.201	0.001					
AX-94511241	6B	30282005	N60, N120 (GY)	1.80E-06	0.202	0.001	TraesCS6B02G050900	30281705	30276637	Protein STRUBBELIG-RECEPTOR FAMILY 5-like	Control male-sterility, organ development, cell proliferation in *Arabidopsis* (Chevalier et al., 2005)
AX-94387975	6B	34398171	N60, N120 (GY)	3.60E-06	0.191	0.001	TraesCS6B02G054200	34341419	34340169	Papain-like cysteine proteinase	Up-regulation of multiple pathogenesis-related proteins and biosynthesis of secondary metabolites (Niño et al., 2020), proteolysis and physiological processes (Liu et al., 2018)
AX-94511284	6B	51737371	N60, N120 (GY)	1.92E-06	0.202	0.001	TraesCS6B02G075400	51736016	51732081	F-box protein At5g03970-like	APO gene in rice improved grain yield per plant (Terao et al., 2010)
AX-94816913	6B	64929420	N60, N120 (GY)	1.33E-06	0.2	0.001	TraesCS6B02G089500	64929918	64925602	F-box protein At5g03970-like	APO gene in rice improved grain yield per plant (Terao et al., 2010)
AX-94607905	2A	571213772	N0, (DSW); N120 (DRW, DSW)	9.13E-07	0.199	0.001	TraesCS2A02G337700	571150423	571149635	Non-specific lipid-transfer protein-like protein At2g13820	Lipid binding and transport, xylem differentiation (Motose et al., 2004; Kobayashi et al., 2011)
AX-94923560	2A	729858414	N60 (FRW,DRW,FSW,DSW)	1.95E-06	0.128	0.001	TraesCS2A02G501900	729858636	729852393	Predicted protein	
AX-94705680	2B	598802253	N120 (DRW); N60 (FSW)	1.29E-06	0.198	0.001	TraesCS2B02G418100	598959096	598956372	acyl-coenzyme A thioesterase 13	acyl-CoA hydrolase activity (Cheng et al., 2006); improves grain filling rate in rice (Zhao et al., 2019); lipid metabolism
AX-95203088	2B	613322090	N60 (FSW,DSW); N120(DRW,DSW)	1.72E-06	0.194	0.001	TraesCS2B02G426600	613234917	613205484	Subtilisin-like protease SBT1.7	Seed coat development and mucilage release (Rautengarten et al., 2008)
AX-94601746	2B	743105753	N60 (FSW), N120 (DRW)	4.85E-07	0.203	0	TraesCS2B02G546300	743331453	743328441	Unnamed protein product	
AX-94887553	2D	580238575	N0 (FSW, DSW); N60 FRW, DRW,DSW); N120 (DRW, FSW)	6.41E-07	0.137	0.001	TraesCS2D02G479500	580238874	580235096	Protein CutA 1, chloroplastic	Cadmium content and leaf margin trait (https://www.uniprot.org/uniprot/Q109R6), copper ion binding (Burkhead et al., 2003); signal transduction (Arnesano et al., 2003); nitrogen regulatory response in bacterial and eukaryotic chloroplast (Ninfa and Atkinsonm, 2000; Arcondéguy et al., 2001)
AX-94735141	2D	581570385	N60 (DRW,DSW, FSW); N120 (FRW,DRW, FSW,DSW)	2.32E-06	0.127	0.001	TraesCS2D02G480200	581526532	581523421	Unnamed protein product	
AX-94474729	2D	584545802	N60 (FRW, DRW)	1.82E-06	0.13	0.001	TraesCS2D02G481800	584545905	584542725	Anthocyanidin reductase	Oxidoreductase activity and flavonoid biosynthetic process (Winkel-Shirley, 2001)
AX-94835810	2D	584799948	N0 (DRW, FSW,DSW); N60 (DRW, DSW); N120 (DRW,FSW,DSW)	2.69E-06	0.175	0.002	TraesCS2D02G482500	584804705	584799573	Predicted protein	
AX-95223893	2D	584861391	N0 (FRW,DRW,FSW,DSW), N60 (DRW,DSW,FSW); N120 (FRW,DRW,FSW, DSW)	3.55E-07	0.186	0.002	TraesCS2D02G482800	584864531	584861189	tRNA (guanine(10)-N2)-methyltransferase homolog	tRNA modification, drought, salt and cold stress 'response, root and plant development in rice and Arabidopsis (Wang et al., 2017)
AX-95003296	2D	586331032	N0 (FSW,DSW); N60 (FRW,DRW)	1.49E-06	0.129	0.001	TraesCS2D02G485600	586331902	586329940	Unnamed protein product	
AX-94477325	2D	586572446	N0 (DRW,FSW,DSW); N60 (FRW,DRW); N120 (DRW)	2.80E-06	0.174	0.002	TraesCS2D02G486000	586575253	586571354	Uncharacterized protein LOC109744903	
AX-95197137	2D	586839201	N0 (FSW, DSW); N60 (FRW, DRW); N120 (DRW,FSW)	7.71E-07	0.133	0.001	TraesCS2D02G486400	586846982	586841634	Receptor-like serine/threonine-protein kinase SD1-8	Regulation of cellular expansion and differentiation in Arabidopsis, ATP and carbohydrate binding, defense and signaling (Uniprot)
AX-94525577	2D	587107149	N0 (FSW,DSW), N60 (FRW,DRW); N120 (FSW)	3.71E-07	0.137	0.001	TraesCS2D02G487000	587107595	587106896	Predicted protein	
AX-94702180	2D	587292781	N0(FSW, DSW); N6 0(FzRW,DRW)	5.41E-07	0.136	0.001	TraesCS2D02G487700	587293147	587282608	Putative kinesin motor domain-containing protein	Regulate rice seed length (Kitagawa et al., 2010); male meiosis, anther dehiscence, and fertility in rice (Zhou et al., 2011)
AX-94487982	2D	588675894	N0 (FSW,DSW); N60 (FRW,DRW); N120 (DRW,FSW)	3.34E-07	0.173	0	TraesCS2D02G489700	588677693	588676152	WRKY45-like transcription factor	Regulates Pi uptake by modulating PHT1;1 expression in Arabidopsis (Wang et al., 2014b); age-triggered leaf senescence (Chen et al., 2017); Benzothiadiazole-inducible blast resistance (Shimono et al., 2007); resistance against *F. graminearum* in wheat (Bahrini et al., 2011); broad-spectrum resistance to wheat powdery mildew (Cao et al., 2011)
AX-95190381	2D	591027900	N0 (FSW,DSW); N60 (FRW, DRW); N120 (DRW,FSW)	2.11E-07	0.18	0	TraesCS2D02G493700	591031752	591027189	Serine-threonine protein kinase	Regulates stress-responsive gene expression in rice (Diédhiou et al., 2008), Negative regulator of immune responses in Arabidopsis (Lin et al., 2015); confers durable and broad-spectrum resistance to wheat powdery mildew (Cao et al., 2011)
AX-95018936	2D	595159320	N60 (DRW); N120 (DRW,DSW)	3.19E-06	0.126	0.001	TraesCS2D02G500500	595161251	595157820	JmjC domain-containing protein	Regulation of RNA silencing, DNA methylation (Qian et al., 2019), Brassinosteroid (BR) signaling pathway, affecting flowering, and biorhythm and bud regeneration (Yokoo et al., 2014)
AX-94457170	2D	596252217	N0 (DSW); N120 (FSW)	2.41E-06	0.164	0.001	TraesCS2D02G502700	596252069	596254635	Adenine nucleotide alpha hydrolases-like superfamily protein	Hydrolase activity and root hair cell differentiation (https://www.uniprot.org/uniprot/Q84JS5); response to salt stress (Jung et al., 2015); involved in male sterility (Mok and Mok, 2001)
AX-94962360	2D	596914793	N0 (DRW,FSW,DSW); N60 (FRW,DRW,DSW); N120 (FSW)	4.51E-07	0.185	0.002	TraesCS2D02G503000	596917719	596911132	Plasma membrane H^+^-ATPase	Plant adaptation to environmental stresses (Janicka-Russak and Kabała, 2015), P deficiency and Al toxicity (Wang et al., 2014a; Yu et al., 2015), transport of various nutrients (nitrate, phosphate and potassium) through roots, elongation of hypocotyls in Arabidopsis (Takahashi et al., 2012); ${\text{NH}}_{4}^{+}$ metabolism in rice roots (Weng et al., 2020); auxin-mediated cell elongation during wheat embryo development (Rober-Kleber et al., 2003)
AX-94829391	2D	601212171	N0 (DSW); N60 (DRW)	1.84E-06	0.128	0.001	TraesCS2D02G507800	601215051	601211912	Nuclease S1	Nucleic acid degradation during plant programmed cell death(Lesniewicz et al., 2013)
AX-94786006	2D	610277424	N60 (FRW,DRW,FSW); N120 (FRW)	1.13E-06	0.131	0.001	TraesCS2D02G521400	610282317	610276851	3-oxoacyl-[acyl-carrier-protein] synthase III, chloroplastic	Fatty acid biosynthesis and metabolism, lipid biosynthesis and metabolism (https://www.uniprot.org/uniprot/P49243), role in rice root development (Ding et al., 2015)
AX-94695716	2D	702726797	N60 (FSW,DSW); N120 (FRW,DRW,DSW)	2.82E-06	0.193	0.002					
AX-95136655	3B	234490336	N0, N60, N120 (DTF)	1.70E-06	0.161	0.016	TraesCS3B02G201300	233224384	233224014	Protein DEHYDRATION-INDUCED 19-like	Drought tolerance in rice (Wang et al., 2014b) and Arabidopsis through up-regulation of pathogenesis-related PR1, PR2, and PR5 gene expressions (Liu et al., 2013); response to salt and water stress (https://www.uniprot.org/uniprot/Q84J70), accelerate flowering (Hwang et al., 2019)
AX-95113687	6A	595578832	N120 (RSA); N60 (Tips)	3.04E-06	0.256	0.006	TraesCS6A02G371000	595564219	595563589	Predicted protein	
AX-94513497	6A	595627899	N0 (RSA, RV); N60 (RSA,RV, Tips); N120 (RSA,RV, Tips)	1.89E-07	0.311	0.001	TraesCS6A02G371300	595628211	595624936	Predicted protein	
AX-94911804	6A	595776559	N0 (RSA, RV); N60 (RSA,RV, Tips); N120 (RSA,RV, Tips, Forks)	5.24E-07	0.307	0.002	TraesCS6A02G371800	595778154	595774917	Vesicle-associated membrane protein 713	Protein transport, response to salt stress (https://www.uniprot.org/uniprot/Q9LFP1), tolerance to water stress (Singh et al., 2017); growth and immune response in Arabidopsis (Yun et al., 2013)
AX-94565231	6D	683646420	N0 (RSA); N60 (RSA, TRL,RV,Tips); N120 (RSA,RV,Forks,Tips)	8.41E-07	0.305	0.003					
AX-94676800	7A	376797697	N60 (FSW); N120 (DRW)	2.79E-06	0.195	0.001	TraesCS7A02G293700	376870968	376869867	Calcium/calmodulin-regulated receptor-like kinase 2	Response to cold, plant tolerance to salt and ABA stress (Yang et al., 2010)

*p-value: significance level of marker-trait association; R^2^: percent phenotypic variance explained by the SNP; FDR: false discovery rate, p-value, R^2^ and FDR is represented as the mean value across seasons*.

**Figure 4:**
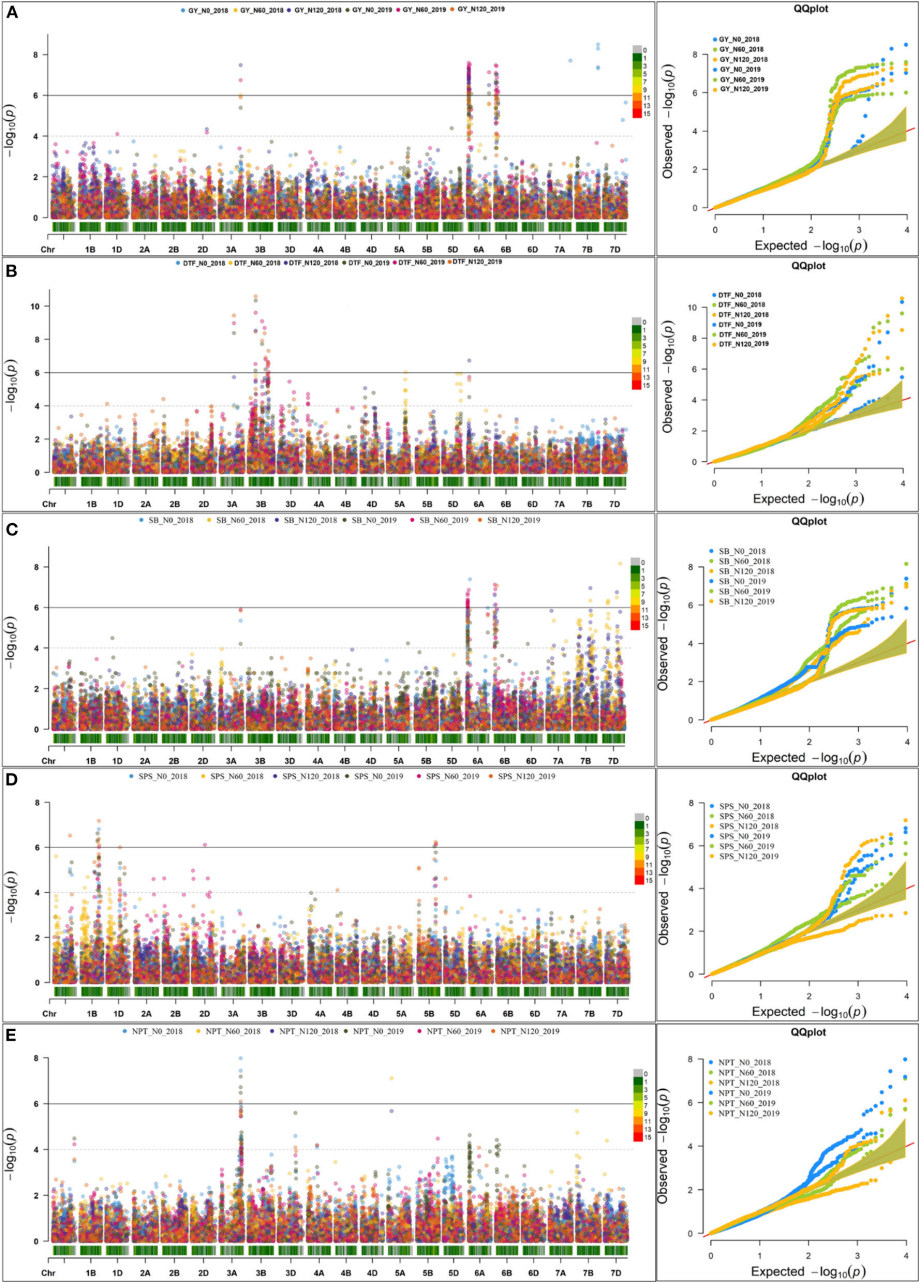
Manhattan plot and qq plot for the yield and yield-related traits across seasons at three different levels of nitrogen (N) (N0, N60, and N120). **(A)** grain yield (GY), **(B)** About 50% days to flowering (DTF), **(C)** shoot biomass (SB), **(D)** spikelets per spike (SPS), and **(E)** number of productive tillers.

**Figure 5:**
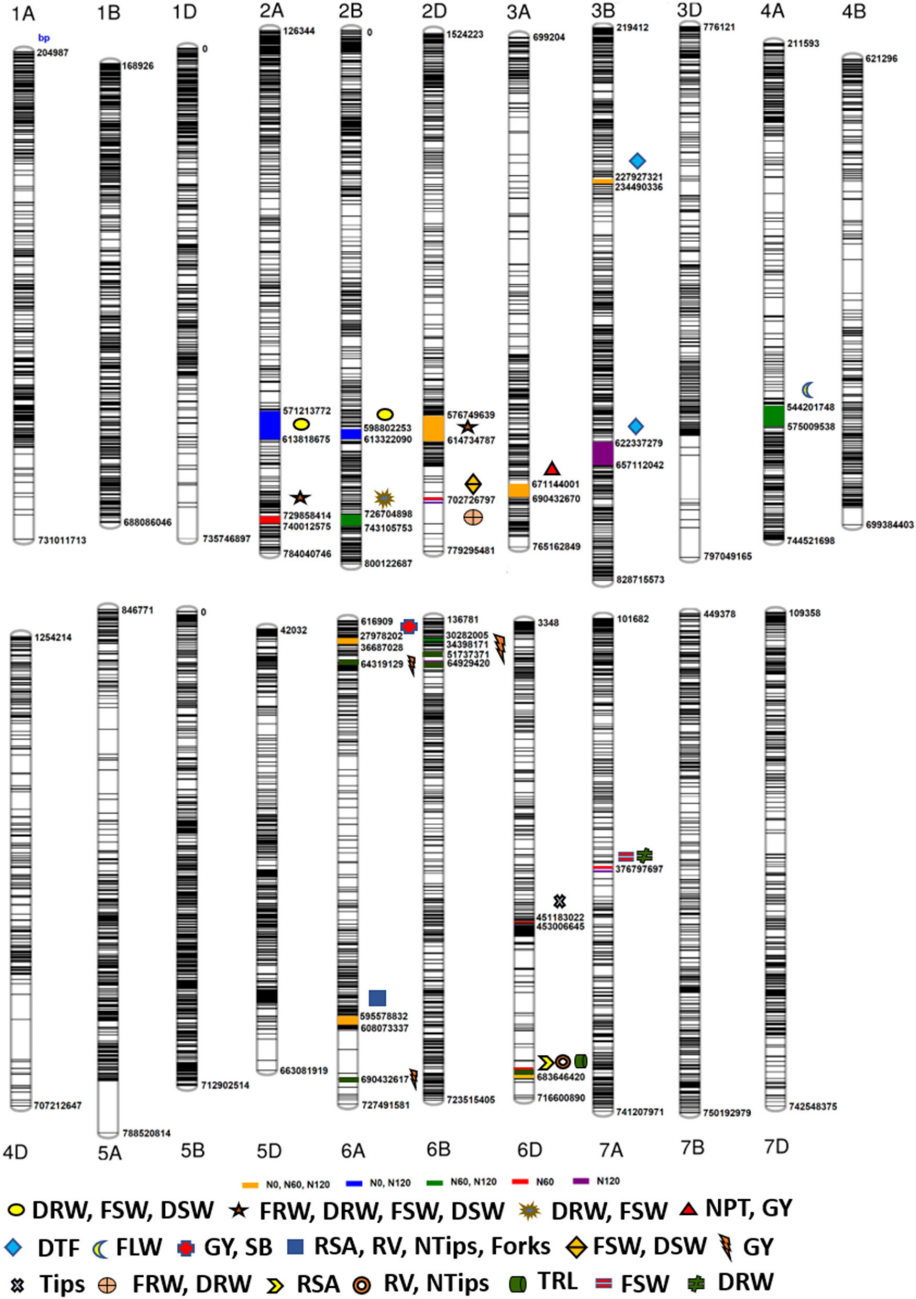
Schematic representation of the single nucleotide polymorphism (SNP) distribution along the 21 chromosomes of wheat. The chromosome map showing genomic regions where marker-trait associations (MTAs) for different nitrogen-use efficiency (NUE)-related trait, root traits, yield, and yield-related traits. The numbers below each chromosome indicate chromosome numbers. The bp represents the physical position of the SNPs on the chromosome in base pair.

**Figure 6:**
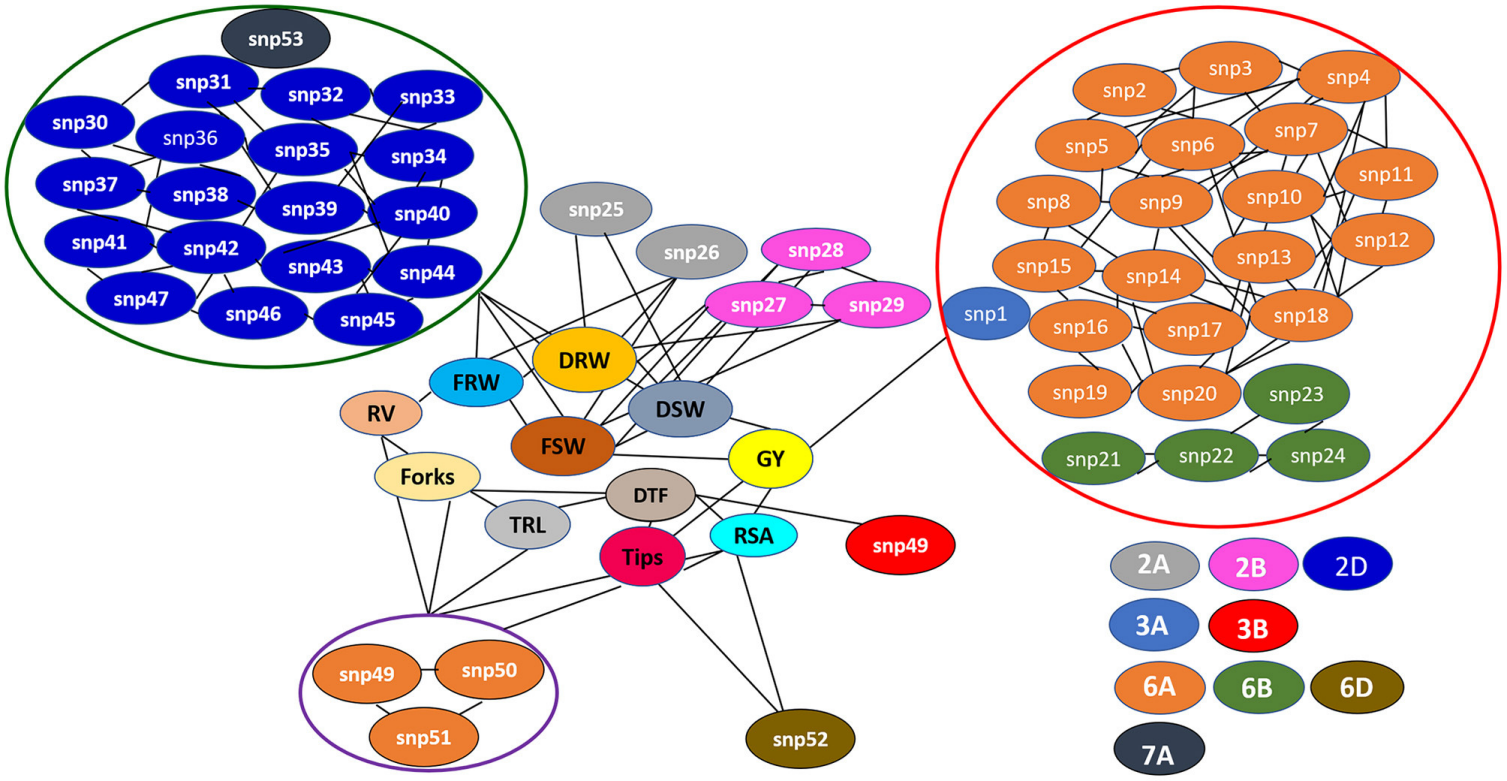
The single nucleotide polymorphism (SNP) network indicating consistent significant marker-trait interactions across seasons and treatments (N0, N60, and N120). *snp1: AX-95136668; snp2: AX-94415776; snp3: AX-94978974; snp4: AX-94737868; snp5: AX-95210745; snp6: AX-95631197; snp7: AX-95011132; snp8: AX-95255669; snp9: AX-95219967; snp10: AX-95070275; snp11: AX-94970334; snp12: AX-94894393; snp13: AX-95249202; snp14: AX-94553503; snp15: AX-94696366; snp16: AX-94386201; snp17: AX-94835065; snp18: AX-94914391; snp19: AX-94606161; snp20: AX-95072891; snp21: AX-94511241; snp22: AX-94387975; snp23: AX-94511284; snp24: AX-94816913; snp25: AX-94607905; snp26: AX-94923560; snp27: AX-94705680; snp28: AX-95203088; snp29: AX-94601746; snp30: AX-94887553; snp31: AX-94735141; snp32: AX-94474729; snp33: AX-94835810; snp34: AX-95223893; snp35: AX-95003296; snp36: AX-94477325; snp37: AX-95197137; snp38: AX-94525577; snp39: AX-94702180; snp40: AX-94487982; snp41: AX-95190381; snp42: AX-95018936; snp43: AX-94457170; snp44: AX-94962360; snp45: AX-94829391; snp46: AX-94786006; snp47: AX-94695716; snp48: AX-95136655; snp49: AX-95113687; snp50: AX-94513497; snp51: AX-94911804; snp52: AX-94565231; snp53: AX-94676800*.

The Manhattan plots depicting the significant -log (*p*-values) for the MTAs associated with nitrogen use efficiency (NUE)-related traits, root traits, and yield/yield related traits measured in the present study at different levels of N are presented in Supplementary Figures 4, 5 and Figure 4, respectively. Location of significant MTAs and SNP marker density distributed across 21 wheat chromosomes is presented in Figure 5. The SNPs for positively correlated traits, such as GY, BY, tips, RSA, RV, and forks appeared to be collocated on Chr 6A at the different levels of N (Table 3). A genomic region on 2D (ranging from 576,749,639 to 702,726,797 bp) contained 25 detected MTAs for a range of traits (FRW, DRW, FSW, and DSW) across seasons and treatments (Table 3 and Figure 5). A cluster of 17 SNPs spanning a 7.7 Mb region on the short arm of 6A showed association with GY at N60 and N120 (Figure 5). Across seasons and treatments, significant association in a 198 kb region on the long arm of Chr 6A were detected for root traits (RSA, RV, tips and forks). The SNP, AX-94565231 at 683.64 Mb on the long arm of Chr 6D showed association with different root traits (RSA, RV, tips, and forks) across seasons and treatments. In the N60 treatment, significant associations for FLW were detected in a 5.6 Mb region (549,799,824–544,201,748 bp) on the long arm of 4A. Interestingly, the association of the trait, DTF with SNP AX-95136655 on Chr 3B at 234.49 Mb was common under N0, N60, and N120 treatments (Figure 5). In the N0 treatment, a significant association harboring three strongly associated SNPs (AX-94593608, AX-94786978, and AX-95134564) spanning the genomic region 76 bp on the long arm of 3A was detected for NPT (Table 3). Further, single SNPs were identified in association with different traits at different N levels. For example, the SNP AX-94914391 (36.43 Mb, 6A) was significantly associated with SB at N0 and with GY at both N60 and N120 (Figure 5). The SNP AX-94705680 (598.80 Mb, 2B) showed association with FSW at N60 and with DRW at N120. The SNP network indicating the significant marker-trait interactions is presented in Figure 6.

### Candidate Gene Identification and Functional Annotation

In order to identify candidate genes underlying the consistent MTAs, we surveyed putative candidates in a 1 Mb upstream and 1 Mb downstream region the identified significant SNPs using EnsemblPlants (http://plants.ensembl.org/index.html). Detailed information on the identified candidate genes is presented in Table 3.

The GO term of identified putative candidate genes was categorized into four groups according to their trait relatedness; The NUE uptake-related (LCC, SPAD, FSW, DSW), root morphological [maximum root length (MRL), TRL), RSA, root diameter (RD), RV, NF, N tips, FRW, and DRW], plant morphological [flag leaf length (FLL), FLW, plant height (PHT)] and GY/yield-attributing traits (days to flowering (DTF), SPS, NPT, SB, and GY]. Most of the putative candidate genes in NUE-uptake related traits across treatments were associated with protein phosphorylation/proteolysis, recognition of pollen, molybdoprotein cofactor biosynthetic process, and transmembrane transport (Supplementary Table 6). Some were part of the cellular component organization and molecular functions of binding molecules and ions, catalytic activity, peptidase activity, and transmembrane transport activity (Supplementary Table 6 and Supplementary Figure 6). The putative candidate genes for the root morphological traits were associated with N compound metabolic processes, phosphorylation, proteolysis, catabolic processes, response to stresses, and regulation of flower development by delineating the composition, and architecture of gene regulatory network underlying flower development and carbohydrate metabolism (Supplementary Table 6 and Supplementary Figure 7). The cellular components include chloroplast, ribosome, membrane, cytoplasm, nucleus, and mitochondria (Supplementary Tables 6, 7). The primary molecular functions related to these genes were catalytic activity (protease, peptidase, hydrolase, transferase, ligase, and oxidoreductase), and binding activity (small molecule binding, ion binding, lipid binding, and carbohydrate derivative binding) (Supplementary Table 6 and Supplementary Figure 7). The putative candidate genes for the plant morphological traits were mainly associated with phosphorylation, response to light-intensity, stress-related responses, and metabolic processes. They were related to the molecular functions of metal ion binding, catalytic activity, kinase activity, and DNA/RNA/ATP binding (Supplementary Table 6 and Supplementary Figure 8). The yield and yield-attributing traits-related putative candidate genes were associated with phosphorylation, metabolic process, protein folding, catabolic process, response to water-stress and light, flower development, and pollen recognition (Supplementary Table 6 and Supplementary Figure 8). The molecular functions include catalytic activity (peptidase, hydrolase, lyase, oxidoreductase, and transferase), binding activity (ion, metal, ATP/GTP, polysaccharide, protein, and DNA), and metabolic activity (Supplementary Table 6 and Supplementary Figure 9).

### Selection of Promising Lines With Stable Performance for Use in Breeding

**Figure 7:**
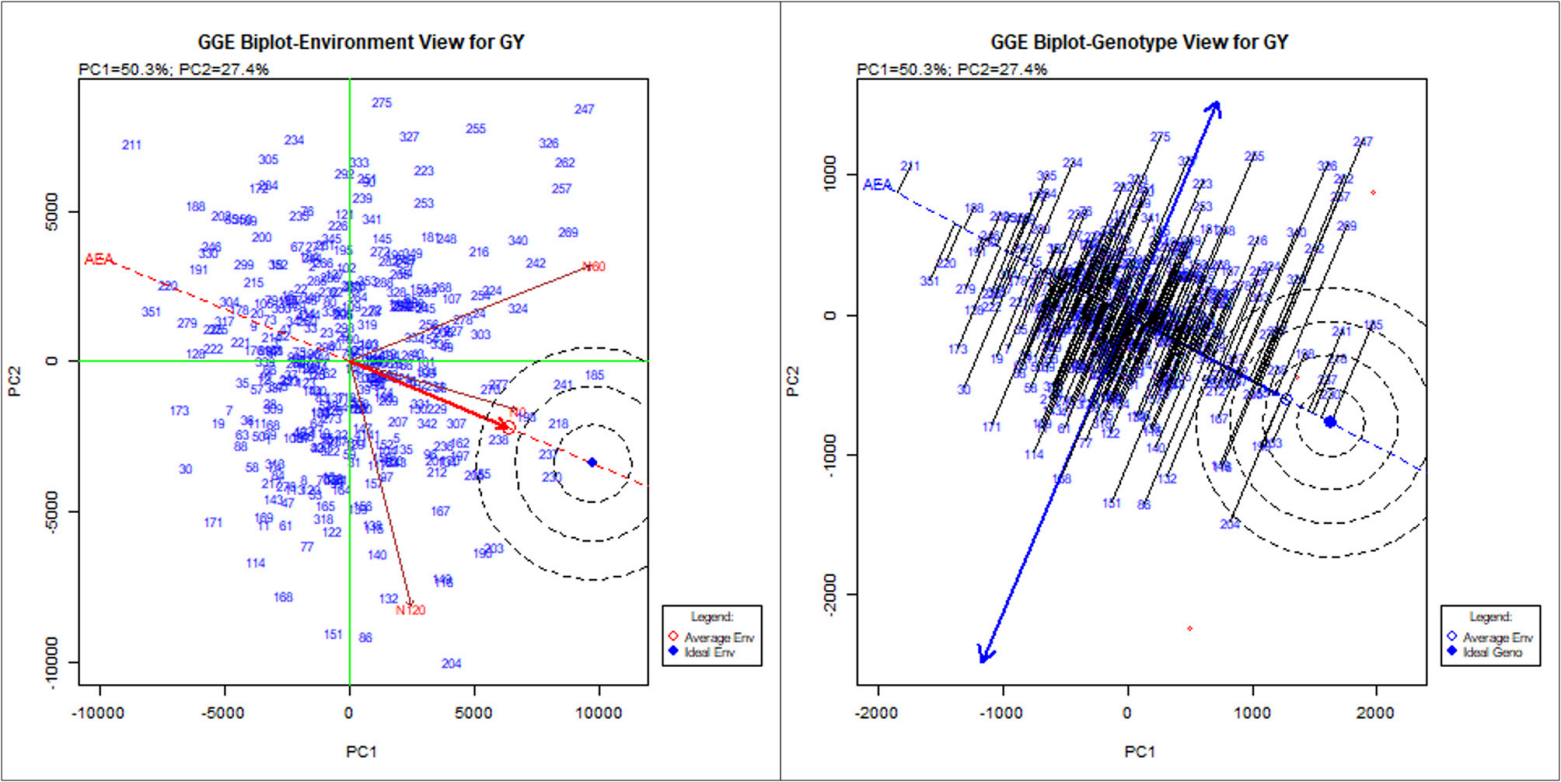
The GGE biplot showing the performance of 352 nested synthetic wheat introgression lines across seasons and treatments (N0, N60, and N120). The environment view refers to the three different levels of nitrogen (N) application: N0, N60, and N120. The genotype view refers to the 352 nested synthetic wheat introgression lines. The numeric number refers to the coding for the introgression lines, which is given in detail in the Supplementary Table 7.

**Figure 8:**
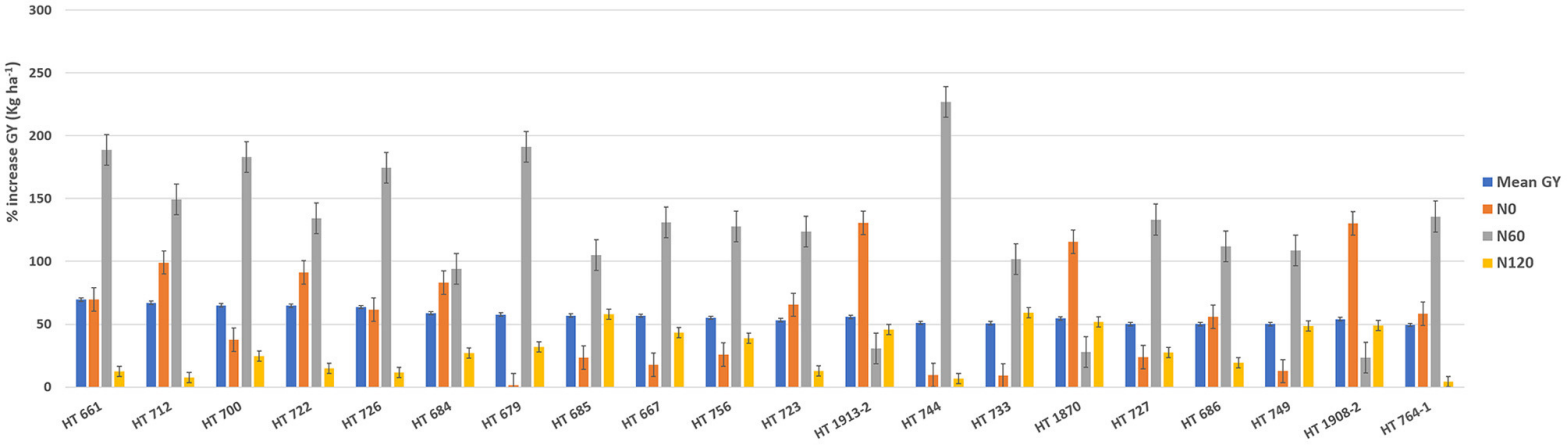
The percentage increase in the grain yield (GY) of top 20 breeding lines derived from the nested introgression libraries possessing high and stable GY (kg ha^−1^) compared to the respective recipient parent averaged across two seasons under three different nitrogen (N) treatments. The numeric values above the bar graph indicate the mean GY (kg ha^−1^) performance of breeding lines across seasons.

**Figure 9:**
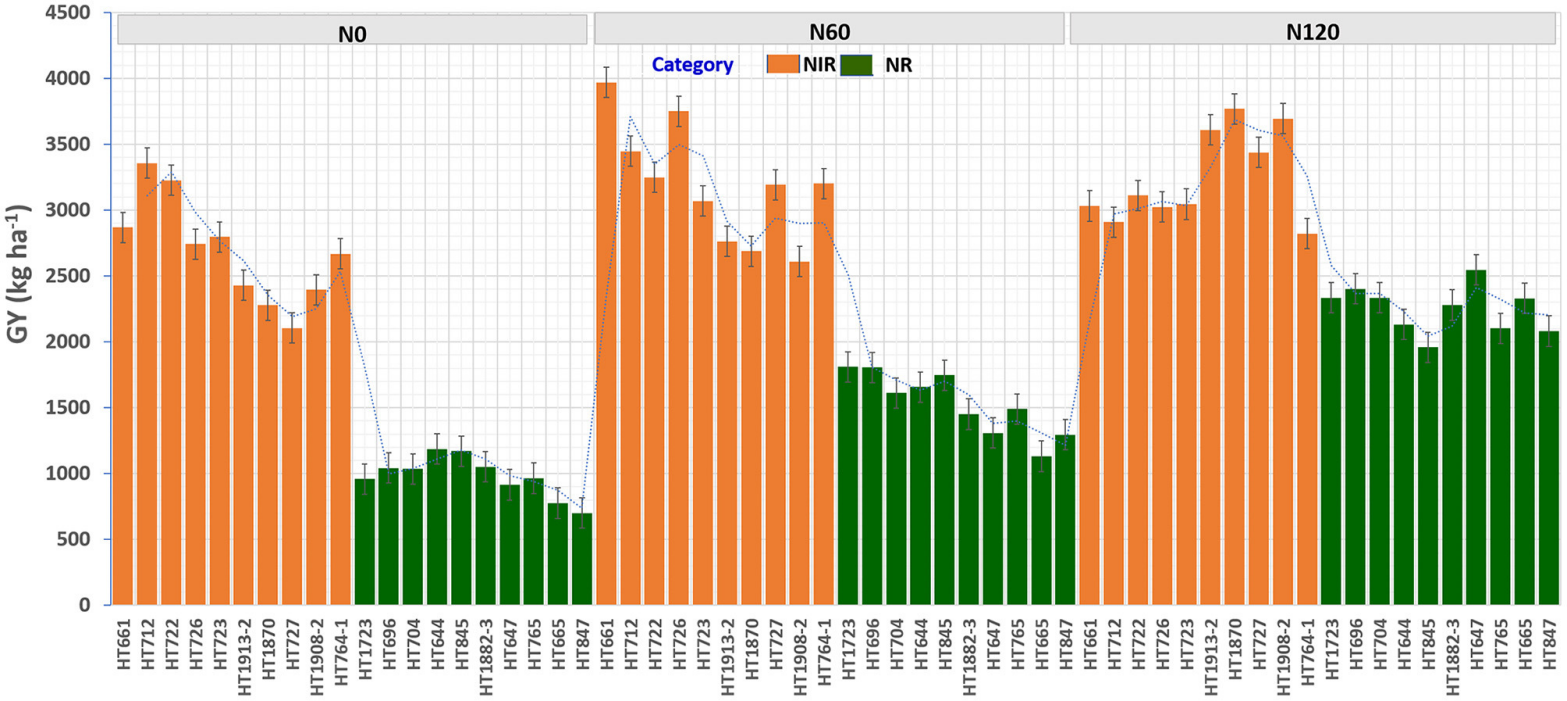
The grain yield (GY) (kg ha^−1^) performance of top 10 nitrogen irresponsive (NIR) and 10 nitrogen responsive (NR) breeding lines averaged over two seasons under three N treatments.

To identify stable breeding lines across treatments and seasons, a GGE biplot method was used. The first two principal components (PCs) explained 77.7% (PC1 = 50.3%, PC2 = 27.4%) of the total GGE variation in the data (Figure 7). The ranking of breeding lines based on their mean GY and stability across seasons and treatments (Supplementary Table 7) was used to identify 20 breeding lines with high and stable yields across seasons and treatments (Supplementary Table 8). The percentage increase in the GY of top 20 breeding lines derived from the nested introgression libraries possessing high and stable GY (kg ha^−1^) compared to the respective recipient parent averaged across two seasons under three different N treatments is presented in Figure 8. Based on GY data across seasons and treatments, the top 10 N-irresponsive (NIR-top grain yielders) and 10 N-responsive (NR-poor grain yielders) breeding lines were also identified (Table 4 and Figure 9).

**Table 4:** The top 10 nitrogen irresponsive (NIR) and 10 nitrogen responsive (NR) breeding lines with contrasting grain yield (GY; kg ha^−1^) derived from pooled mean over two seasons and three treatments.

**Category**	**Designation**	**Pop**	**Mean GY**	**2018_N0**	**2019_N0**	**2018_N60**	**2019_N60**	**2018_N120**	**2019_N120**
NIR	HT661	Pop1	3289	2865	2869	4093	3843	3190	2873
NIR	HT712	Pop1	3237	3145	3568	3435	3458	2902	2915
NIR	HT722	Pop1	3195	3102	3349	3158	3338	2892	3328
NIR	HT726	Pop1	3172	2982	2502	3978	3522	2845	3203
NIR	HT723	Pop1	2970	2802	2791	3207	2930	3222	2867
NIR	HT1913-2	Pop4	2933	2332	2526	2909	2616	3693	3523
NIR	HT1870	Pop4	2911	2122	2433	2632	2745	3665	3871
NIR	HT727	Pop1	2911	2377	1833	3370	3012	3610	3266
NIR	HT1908-2	Pop4	2900	2641	2148	2851	2368	3553	3837
NIR	HT764-1	Pop2	2897	2440	2897	3528	2872	2747	2896
NR	HT1723	Pop3	1885	1158	760	1806	1813	2675	3096
NR	HT696	Pop1	1750	825	1261	1660	1950	2455	2350
NR	HT704	Pop1	1661	827	1245	1948	1274	2297	2372
NR	HT644	Pop1	1658	989	1384	1838	1474	1822	2443
NR	HT845	Pop2	1625	1111	1232	1700	1793	2038	1878
NR	HT1882-3	Pop4	1594	918	1186	1256	1646	1982	2576
NR	HT647	Pop1	1590	846	985	1082	1535	2440	2652
NR	HT765	Pop2	1519	768	1162	1375	1606	1720	2484
NR	HT665	Pop1	1413	542	1010	1187	1077	2370	2292
NR	HT847	Pop2	1359	683	717	1258	1333	1980	2182

**Figure 10:**
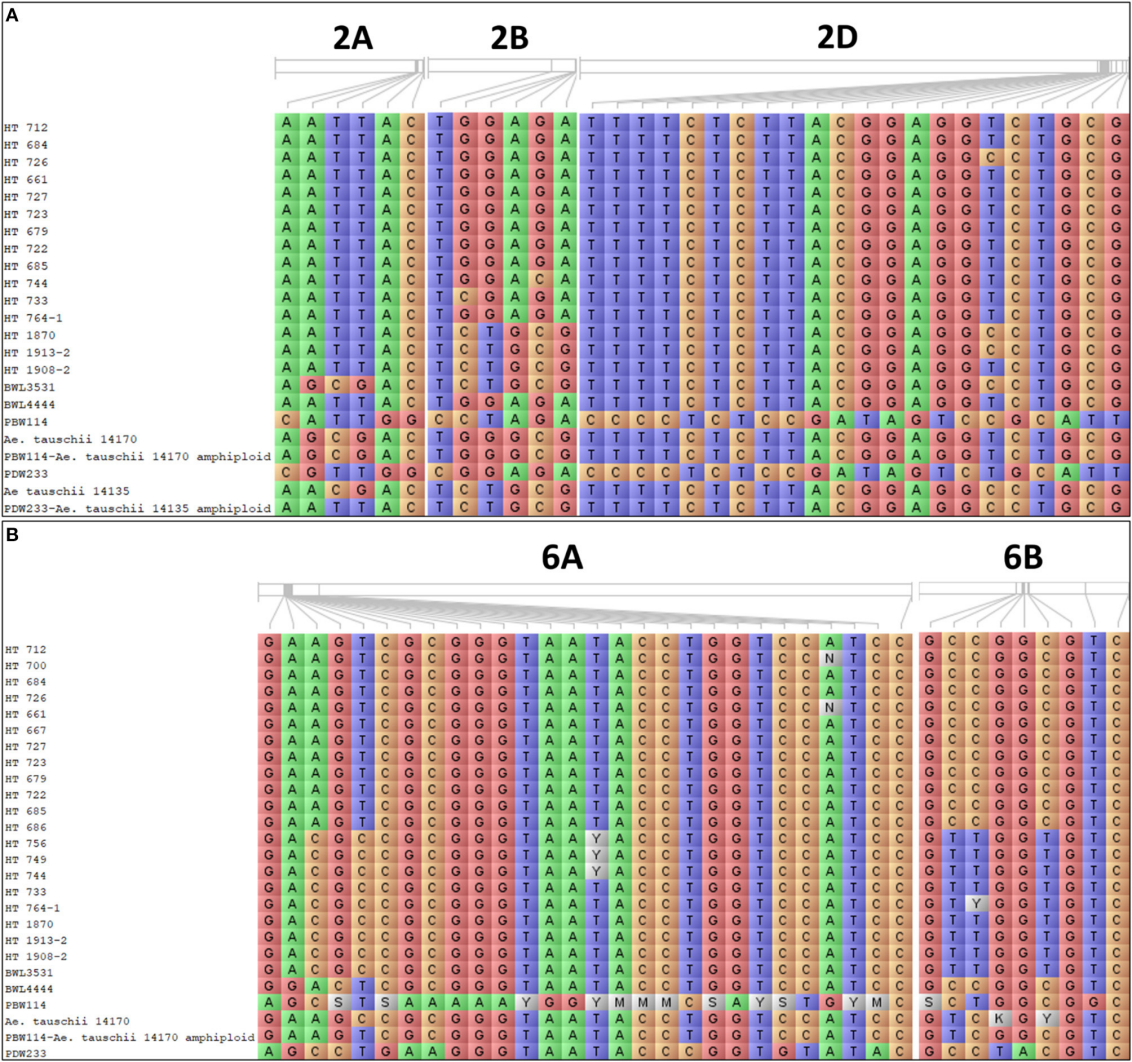
The allelic constitution of the selected promising breeding lines, wild accessions of *Ae. tauschii*, cultivated and synthetic wheats for the **(A)** root-related traits and **(B)** grain yield (GY).

Further analysis was undertaken to assess the significant differences between the mean values of the allelic classes of MTAs for root growth and GY using the Kruskal–Wallis test. The presence of favorable alleles with significant differences was checked in promising breeding lines. This allowed for the selection of 20 promising breeding lines possessing favorable allele combinations for improving the plant root growth (Figure 10A) and grain GY under N limitation (Figure 10B).

## Discussion

An increase in crop production by the development of high-yielding varieties is largely dependent on the supply of N fertilizers. Excessive application of nitrogenous fertilizer is becoming very expensive which accounts for the great loss of economic profit to the farmers in addition to the negative impacts on the environment (Hawkesford and Griffiths, 2019). The reliable phenotyping under low nitrogen input is very challenging and affected by genotype (G), environment (E), and the G x E interactions (Rao et al., 2018). Proper understanding of the genotype behavior, identification, and development of N-efficient genotypes without compromising the GY is a paramount need for improving the NUE. Notably, very few wheat breeding programs are targeting the development of N-efficient genotypes. In crop plants, such as wheat, the efforts are constrained due to a lack of variation in the cultivated germplasm for NUE. The narrow genetic diversity and fewer recombination events in the biparental mapping populations may result in poor quantitative trait loci (QTL) detection power (Gangurde et al., 2019). The next-generation high-resolution mapping populations, such as nested synthetic wheat introgression libraries used in the present study may provide a vast and untapped source of genetic variations for the NUE-related traits due to high numbers of recombination events. The use of synthetic hexaploid wheat in the present study presents an effective genetic resource for transferring the agronomically important genes from wild relatives to the common wheat. The introgression of favorable alleles associated with root traits and GY from *Ae. tauschii* wild accessions to cultivated wheat (Figure 10) indicated the potential of synthetic wheat providing new sources for improving yield potential and NUE when bred with the modern wheat varieties.

The different traits associated with N uptake and NUE were studied in nested synthetic wheat introgression libraries at three different nitrogen levels. The ANOVA results revealed the native variation across the genotypes toward the N response which had given the possibility to identify the NUE lines under different levels of N. The genotypic variations purely reveal the phenotypic plasticity of the breeding lines toward traits. The diverse responses have been observed among the breeding lines across different levels of N, despite similar growth conditions and an equal amount of nitrogenous fertilizer application in a given N level as indicated by significant differences among the genotypes within and across treatments and non-significant differences among the replications. Significant G x E, G x S, G x E, and G x T x S interactions indicated that the seasons and environments under different levels of N application were a critical factor in explaining the genotypic variance for the traits measured in the present study. The results reported in the present study concurred with other reported studies on rice (Srikanth et al., 2016) and wheat (Sial et al., 2005; Belete et al., 2018).

In general, the increase in GY was correlated with the increase in the rate of N fertilizer application, which might be due to the availability of sufficient N for the proper growth and development of the plants. Šarčević et al. (2014) reported a 10% reduction of GY at low N condition compared to normal condition in wheat. The significant and positive correlation among different root traits and GY and yield-attributing traits indicated complementary functional roles of the root traits in improving the GY by improving the nutrient acquisition from the soil. The collocation of MTAs for the correlated traits strengthens the significance of MTAs. A significant positive correlation between GY and NUE-related traits in wheat, maize, and oilseed rape (Fageria et al., 2010; He et al., 2017; Belete et al., 2018) signified the importance of NUE-related traits in improving the GY under limited N conditions.

Different mapping approaches using nested-association mapping (NAM) populations successfully exploited the genetics of complex traits and facilitated the discovery of candidate genes in rice (Fragoso et al., 2017), wheat (Hu et al., 2018; Jordan et al., 2018), maize (Yu et al., 2008; McMullen et al., 2009) and soybean (Song et al., 2017; Xavier et al., 2018). For NUE-related traits, significant genetic variations in hybrids, open-pollinated populations, large germplasm panels, backcross, and recombinant inbred line populations in different cereal crops, such as rice, wheat, maize, and oilseed rape were observed (Chen et al., 2014; Li et al., 2015; Vijayalakshmi et al., 2015; Ertiro et al., 2017; He et al., 2017; Rao et al., 2018). Mapping for NUE -related traits using different populations and mapping approaches highlight the complex nature of the trait.

In the present study, the nested synthetic wheat introgression libraries were designed for the identification of genomic regions associated with traits related to NUE using genome-wide association study (GWAS) keeping into account, the genetic effects produced in each genetic background. The associated SNPs were used to track the potential candidate genes associated with a particular trait of interest. The presence of high phenotypic variability in the nested synthetic introgression libraries coupled with the high marker density across the whole genome provided a strong base for the association mapping.

Interestingly, the genes responsive to nutrient uptake under water stress (Diédhiou et al., 2008; Janicka-Russak and Kabała, 2015; Wang et al., 2017), shoot growth, root and plant development (Wang et al., 2017), nutrient uptake and transport of various nutrients (Takahashi et al., 2012; Wang et al., 2014b; Weng et al., 2020) reported to be collocated with 126 Mb genomic region on Chr 2D constituting 25 MTAs which stood out as hot-spot for different traits (FRW, DRW, FSW, and DSW) in the present study. This indicates the positive interactions between root traits, nutrient uptake, and plant growth and development. The 7.7 Mb region on the short-arm of Chr 6A constituting 17 SNPs associated with GY showed collocation with the genes that were directly or indirectly involved in improving the GY in different cereal crops. These include the genes controlling flowering (Kania et al., 1997), panicle, and seed development (Jain et al., 2007; Li et al., 2011), GY (Terao et al., 2010), resistance to pathogenesis (Taniguchi et al., 2014; Wang et al., 2015; Niño et al., 2020) and abiotic stress tolerance (Brands and Ho, 2002; Palusa et al., 2007). The MTAs associated with different root traits, such as RSA, RV, tips, and forks in the present study were located near the earlier reported genes involved in regulating abscisic acid sensitivity and root growth development in Arabidopsis (Rodriguez et al., 2014) and adaptation under water stress conditions in wheat (Singh et al., 2017). Interestingly, the gene accelerating flowering in Arabidopsis (Hwang et al., 2019) was observed to be collocated with the SNP AX-95136655 associated with DTF on Chr 3B in the present study. The collocation of identified MTAs with earlier reported genes controlling the photosynthetic traits, root development, plant growth, nutrient uptake and transport, flowering, resistance to pathogenesis, and stress-responsive genes further confirms the contribution of these identified traits/MTAs in improving the N uptake/utilization and GY under N-limited conditions. The identified nitrogen irresponsive (NIR) breeding lines with favorable alleles in combination with the multiple traits might serve as potential donors for the development of N-efficient wheat varieties.

## Conclusions

The nested synthetic introgression libraries covering extensive phenotypic variability coupled with huge genome coverage were used to identify the significant MTAs associated with nitrogen-use efficiency (NUE)-related traits in wheat. Significant phenotypic variations for the NUE-related traits, yield, and yield-related traits among genotypes, treatments, seasons, and their interactions (genotype x treatment, genotype x season, treatment x season, and genotype x treatment x season) were observed. Stable marker-trait associations (MTAs) identified for different traits measured in the present study comigrating with various genes associated with nitrogen (N) uptake/utilization and improving grain yield (GY) may help to harness their benefits in genomics-assisted breeding programs. The identification of N-efficient breeding lines may serve as novel donors in genomics-assisted introgression programs. The identification and introgression of superior haplotype improving NUE while maintaining GY using haplotype-based breeding may open new avenues in designing next-generation N-efficient high-yielding wheat varieties.

## Data Availability Statement

The original data and related information presented in the study are included in the article/Supplementary Material, further inquiries can be directed to the corresponding author.

## Author Contributions

NS and PC designed this study. AK provided the genotypic data of two populations and contributed to the development of nested introgression libraries. NS and MS conducted the field experiments. NS analyzed the data and wrote the manuscript. NS, SK, and PC provided resources. AK, MS, SK, VP-S, AS, AB, TB, and PC revised the manuscript. VP-S provided support with SPAD meter. All authors contributed to the article and approved the submitted version.

## Funding

The work was compiled under the projects funded by the Department of Biotechnology, Govt. of India (Grant No. BT/IN/UK-VNC/42/RG/2015-16 and BT/PR30871/BIC/101/1159/2018).

## Conflict of Interest

The authors declare that the research was conducted in the absence of any commercial or financial relationships that could be construed as a potential conflict of interest.

## Publisher's Note

All claims expressed in this article are solely those of the authors and do not necessarily represent those of their affiliated organizations, or those of the publisher, the editors and the reviewers. Any product that may be evaluated in this article, or claim that may be made by its manufacturer, is not guaranteed or endorsed by the publisher.
